# Molybdenum Diphosphide Nanorods with Laser‐Potentiated Peroxidase Catalytic/Mild‐Photothermal Therapy of Oral Cancer

**DOI:** 10.1002/advs.202101527

**Published:** 2021-10-31

**Authors:** Min Qian, Ziqiang Cheng, Guanghong Luo, Massimiliano Galluzzi, Yuehong Shen, Zhibin Li, Hongyu Yang, Xue‐Feng Yu

**Affiliations:** ^1^ Department of Oral and Maxillofacial Surgery Guangdong Provincial High‐level Clinical Key Specialty Guangdong Province Engineering Research Center of Oral Disease Diagnosis and Treatment Peking University Shenzhen Hospital Guangdong 518036 P. R. China; ^2^ Materials and Interfaces Center Shenzhen Institute of Advanced Technology Chinese Academy of Sciences Shenzhen Guangdong 518055 P. R. China; ^3^ Department of Radiation Oncology The Second Clinical Medical College Jinan University (Shenzhen People's Hospital) Shenzhen Guangdong 518020 P. R. China

**Keywords:** chemodynamic therapy, mild‐photothermal therapy, molybdenum diphosphide nanorods, oral cancer, peroxidase‐like catalytic

## Abstract

Chemodynamic therapy (CDT) is an emerging treatment that usually employs chemical agents to decompose hydrogen peroxide (H_2_O_2_) into hydroxyl radical (•OH) via Fenton or Fenton‐like reactions, inducing cell apoptosis or necrosis by damaging biomacromolecules such as, lipids, proteins, and DNA. Generally, CDT shows high tumor‐specificity and minimal‐invasiveness in patients, thus it has attracted extensive research interests. However, the catalytic reaction efficiency of CDT is largely limited by the relatively high pH at the tumor sites. Herein, a 808 nm laser‐potentiated peroxidase catalytic/mild‐photothermal therapy of molybdenum diphosphide nanorods (MoP_2_ NRs) is developed to improve CDT performance, and simultaneously achieve effective tumor eradication and anti‐infection. In this system, MoP_2_ NRs exhibit a favorable cytocompatibility due to their inherent excellent elemental biocompatibility. Upon irradiation with an 808 nm laser, MoP_2_ NRs act as photosensitizers to efficiently capture the photo‐excited band electrons and valance band holes, exhibiting enhanced peroxidase‐like catalytic activity to sustainedly decompose tumor endogenous H_2_O_2_ to •OH, which subsequently destroy the cellular biomacromolecules both in tumor cells and bacteria. As demonstrated both in vitro and in vivo, this system exhibits a superior therapeutic efficiency with inappreciable toxicity. Hence, the work may provide a promising therapeutic technique for further clinical applications.

## Graphical abstract

A promising therapeutic strategy of laser‐potentiated peroxidase catalytic/mild‐photothermal therapy of molybdenum diphosphide nanorods (MoP_2_ NRs) is proposed to improve CDT performance and achieve effective tumor eradication and anti‐infection. In this system, MoP_2_ NRs exhibit favorable cytocompatibility and peroxidase‐like catalytic activity to decompose tumor endogenous H_2_O_2_ into •OH radicals, which subsequently break biomolecules, simultaneously inducing tumor cells apoptosis and bacteria inactivation.

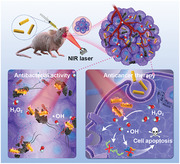

## 1 Introduction

Oral cancer is a major global health issue with more than 350 000 newly diagnosed cases per year with associated mortality rate reaching around 45%.^[^ ^1^, ^2^, ^3^, ^4^, ^5^ ^]^ Despite early detection is helping to improve the 5‐year survival rate of patients,^[^ ^6^, ^7^ ^]^ there are approximately half of diagnosed cases staged III or IV.^[^ ^8^ ^]^ At those stages, the metastasis has already occurred. The routinely delayed diagnosis results in poor quality of life in patients. The available gold standard treatments for oral cancer, including surgery, radiation and chemotherapy generally fail to completely eradicate tumor while causing severe side effects,^[^ ^9^, ^10^, ^11^ ^]^ such as the undesirable esthetic impairment of oral cavity and face, systemic drug toxicity, and immunological responses.^[^ ^12^, ^13^, ^14^, ^15^ ^]^ Moreover, the microbial floras in the oral cavity are rich and diverse,^[^ ^16^, ^17^, ^18^ ^]^ and the commonly used cancer therapies are prone to cause severe infections, which are usually considered as the critical risk factors associated with postoperative complications inducing morbidity and mortality.^[^ ^19^, ^20^ ^]^ Therefore, there is an urgent need to develop a minimally or non‐invasive technology that simultaneously achieves effective tumor eradication and anti‐infection mechanism offering a potent therapeutic effect.

Recently, chemodynamic therapy (CDT),^[^ ^21^, ^22^ ^]^ as an emerging cancer therapy strategy, has attracted tremendous interest. The method uses chemical agents to selectively catalyze the endogenous hydrogen peroxide (H_2_O_2_) (concentration range from 100 µM to 1 mm) of tumors in hydroxyl radical (•OH).^[^ ^23^, ^24^, ^25^ ^]^ Generally, transition metal ions such as Fe^2+^, Mn^2+^, Cu^1+^, and V^2+^ are employed to catalyze the decomposition of H_2_O_2_.^[^ ^24^, ^26^ ^]^ In this reaction, H_2_O_2_ can be reduced to •OH, one of the most powerful reactive oxygen species (ROS) via Fenton/Fenton‐like reactions,^[^ ^27^, ^28^ ^]^ thus eliciting cell apoptosis or necrosis.^[^ ^28^, ^29^, ^30^, ^31^ ^]^ Moreover, •OH species can damage bacterial biomacromolecules, including membrane lipids, cellular proteins, and DNA, finally, resulting in efficient bacterial inactivation.^[^ ^32^, ^33^, ^34^ ^]^ Owing to the higher tumor specificity and minimal side effects,^[^ ^26^ ^]^ CDT nanomaterials have good potential applications in oral cancer therapy. Nevertheless, some challenges still remain: 1) The catalytic reaction efficiency of CDT agents is restricted in the tumor microenvironment, wherein the concentration of endogenous H_2_O_2_ is low and the pH value is relatively high for Fenton/Fenton‐like reactions, influencing the amount of •OH generation and ultimately affecting the therapeutic efficiency;^[^ ^35^, ^36^ ^]^ 2) limited by the low catalytic reaction efficiency,^[^ ^37^, ^38^ ^]^ large doses of CDT agents are generally administered to achieve a sufficient therapeutic effect, which may cause a burst‐type release of CDT molecules in short‐time period and thereby causing undesirable side effects such as nephrotoxicity or severe allergic reactions. Thus, these limitations prompted us to develop new strategies to improve the therapeutic efficacy of CDTs and seek more appropriate CDT agents for further clinical translations. In this work, we successfully fabricated biocompatible CDT agents composed of MoP_2_ nanorods (NRs), carefully explored their potential as robust CDT agents, which has not been investigated up to date.

**Scheme 1:**
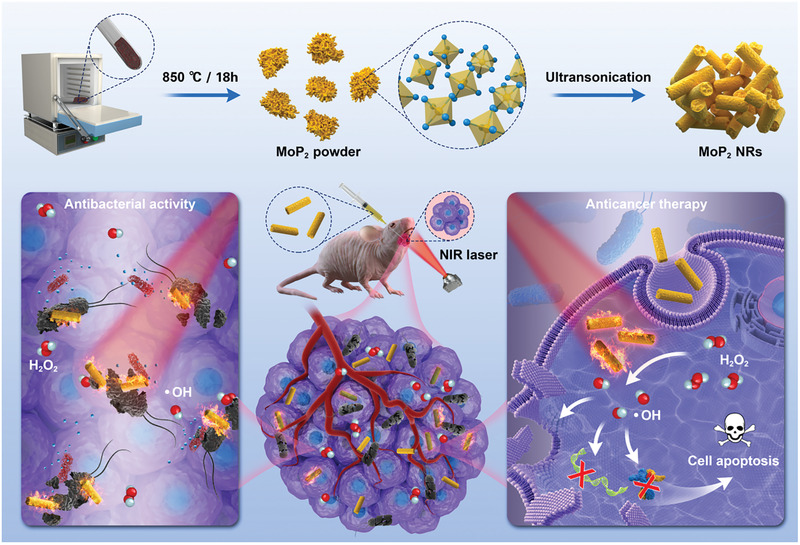
The schematic diagram illustrates the fabrication of MoP_2_ NRs and their biomedical application as a laser‐potentiated peroxidase catalytic/mild‐photothermal agent, simultaneously achieving effective tumor eradication and anti‐infection in oral cancer therapy.

Considering their semimetal nature, MoP_2_ NRs have unique properties, such as small effective mass, high carrier mobility, and very small band overlap energy,^[^ ^39^, ^40^ ^]^ recently attracting extensive interest in applications for optoelectronics. However, previous research efforts were mainly focused on their physical properties and photocatalytic activities,^[^ ^41^, ^42^, ^43^ ^]^ while the biomedical applications have rarely been explored. In this study, we found that MoP_2_ NRs also possess favorable biocompatibility, intriguing photothermal conversion efficacy, and peroxidase‐like activity.^[^ ^41^, ^44^ ^]^ In particular, MoP_2_ NRs are biodegradable, they can gradually degrade to free Mo ions and phosphates in the in vivo environment. Since Mo is an essential trace element of life, and P is an essential element for maintaining human health, which approximately taking up 1% of the body weight,^[^ ^45^, ^46^, ^47^ ^]^ such unprecedented advantages may enable MoP_2_ NRs to have great promise for future clinical translations. In CDT, MoP_2_ NRs can display peroxidase‐like activity to trigger the decomposition of H_2_O_2_ and generate highly efficient •OH. Interestingly, when combined with near‐infrared light irradiation, the peroxidase‐like activity of MoP_2_ NRs is sharply enhanced. We discovered that laser‐triggered peroxidase catalytic of MoP_2_ NRs increases the therapeutic performance in oral cancer therapy. As illustrated in **Scheme** **1**, MoP_2_ NRs are initially synthesized via a facile liquid exfoliation and collected by centrifugation. The as‐synthesized MoP_2_ NRs show a considerable photothermal conversion efficiency and favorable element biocompatibility in vitro. Upon intratumor injection and assisted by 808 nm laser irradiation, the photo‐excited band electrons and valance band holes are effectively adsorbed by MoP_2_ NRs, consequently consuming the tumor endogenous H_2_O_2_ to sustain the production of •OH radicals. These radicals can disrupt the cell membrane integrity and induce oxidative stress to bring DNA damage, eventually resulting in cell apoptosis and death of bacteria. Compared to CDT or photothermal therapy (PTT) treatment alone, this parallel therapeutic modality remarkably improves the antitumor therapeutic efficiency of CDT and simultaneously prevents the complications caused by bacterial infections. It must be considered that conventional PTT‐induced local hyperthermia usually works over 50 °C,^[^ ^48^ ^]^ a temperature higher than skin tolerance, causing inevitable damage to the surrounding healthy tissues by heat diffusion.^[^ ^49^ ^]^ In our study, we can maintain the temperature at a relatively low level in the tumor sites (≤43 °C) to avoid the unwanted over‐heating by NIR laser.^[^ ^48^ ^]^ Based on these merits, MoP_2_ NRs have potential as a promising agent for laser‐potentiated CDT/PTT tumor eradication and antibacterial performance simultaneously. Our findings provide a novel paradigm for oral cancer therapy and thus may benefit patients in future clinical applications.

## 2 Results and Discussion

### 2.1 Preparation and Characterization of Molybdenum Diphosphide Nanorods

**Figure 1:**
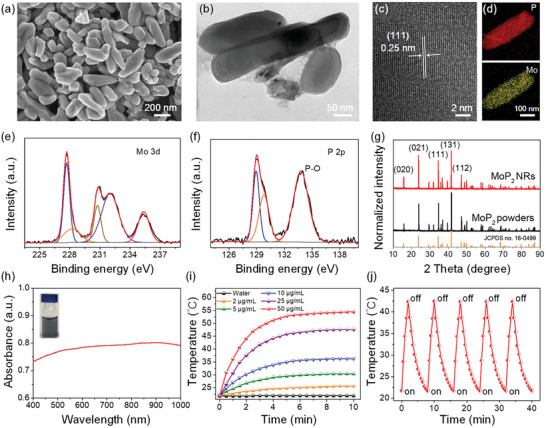
Morphology and characterization of as‐synthesized MoP_2_ NRs. a) SEM image, b) TEM image, c) HR‐TEM image, and d) elemental maps. e) Mo 3d XPS spectra, f) P 2p XPS spectra, and g) XRD patterns of the as synthesized MoP_2_ NRs and their crystals. h) The UV–vis absorption spectrum of MoP_2_ NRs aqueous solution. The inset shows the photograph of MoP_2_ NRs aqueous solution at the same concentration. i) Photothermal heating curves of MoP_2_ NRs solutions with different concentrations (0, 2, 5, 10, 25, 50 µg mL^–1^) under irradiation with an 808 nm laser (1.0 W cm^–2^). j) Temperature variation of the MoP_2_ NRs solution during five laser on/off cycles.

MoP_2_ NRs were prepared through high temperature reaction and ultrasonication‐assisted exfoliation. First, coral‐like MoP_2_ crystals were synthesized by direct reaction of molybdenum and red phosphorus in a quartz tube with high‐vacuum (Figure S1, Supporting Information). Then the MoP_2_ NRs were successfully obtained by probe ultrasonic exfoliation of MoP_2_ crystals in *N*‐methyl‐2‐pyrrolidone (NMP). The crystal phase and purity of the as‐synthesized MoP_2_ NRs were examined by X‐ray powder diffraction (XRD). The morphology of MoP_2_ NRs was visualized by scanning electron microscopy (SEM) and transmission electron microscopy (TEM). As shown in **Figure** **1a**,b, the as‐obtained MoP_2_ NRs have irregular shapes with transverse diameter within ≈100 nm and length from ≈100 to 400 nm. The high‐resolution transmission electron microscopy image in Figure 1c shows distinct lattice fringes with an interplanar spacing of ≈0.25 nm, corresponding to the spacing of the MoP_2_ (111) crystal planes (JCPDS no. 16–0499). Elemental mapping shown in Figure 1d clearly reveals that both molybdenum and phosphorus are homogeneously distributed across the whole nanorod. In addition, X‐ray photoelectron spectroscopy (XPS) was performed to investigate the surface composition and chemical state of MoP_2_ NRs. As depicted in Figure 1e, the Mo 3d spectrum can be resolved into five peaks. The peaks observed at 235.3 and 232.0 eV are corresponding to the typical Mo^6+^ 3d_3/2_ and Mo^6+^ 3d_5/2_ states of MoP_2_, while the rest of the Mo 3d peaks located at 230.7, 228.2, and 227.7 eV are belonging to Mo^*δ*+^ (0 < *δ* ≤ 4).^[^ ^50^, ^51^ ^]^ In the high‐resolution XPS spectrum of P 2p (Figure 1f), doublet peaks at 129.8 (P 2p_1/2_) and 128.9 eV (P 2p_3/2_) can be assigned to P that bonded to Mo in MoP_2_, while the peak at 133.8 eV is associated with the oxidized P species.^[^ ^40^ ^,,^ ^51^, ^52^ ^]^ As shown in Figure 1g, all the sharp diffraction peaks are matched well with the standard pattern of MoP_2_ (JCPDS no. 16–0499), demonstrating the high purity of the as‐prepared MoP_2_ NRs with the absence of any phosphorus or molybdenum by‐products.

The as‐prepared MoP_2_ NRs exhibited good dispersibility in water, the aqueous solution of MoP_2_ NRs showed a greenish‐black color with a broad absorbance band from visible to near‐infrared (NIR) region, specially at 808 nm (Figure 1h). Meanwhile, the stability of MoP_2_ NRs in a variety of media (water, PBS, DMEM, and DMEM supplemented with 10% FBS) was monitored by a digital camera. As shown in Figure S2, Supporting Information, MoP_2_ NRs were well‐dispersed in the relevant media with a discrete state. After 8 h incubation, the degradation and aggregation were observed, indicating the excellent biodegradability of MoP_2_ NRs. Furthermore, the biodegradation of MoP_2_ NRs was further investigated and confirmed by the corresponding visual inspection and UV–vis–NIR absorption spectra (Figure S3, Supporting Information). In view of the Beer‐Lambert Law (Figure S4, Supporting Information), the mass extinction coefficient of MoP_2_ NRs was calculated to be 20.5 L g^–1^ cm^–1^ at 808 nm, representing 5.26‐fold higher than that of Au nanorods (3.9 L g^–1^ cm^–1^).^[^ ^53^ ^]^ In addition, the photothermal transduction efficiency (*η*) of MoP_2_ NRs was calculated to be 18.8% (Figure S5, Supporting Information), and their photothermal performance in water was further examined. The photothermal curves demonstrated a prominent concentration‐/laser power‐dependent temperature increases (Figure 1i and Figure S6, Supporting Information), upon a 10‐min irradiation, the temperature of solutions rapidly increased within 2 min, while pure water did not show an apparent temperature‐rising under the same conditions. In addition to the excellent photothermal performance, MoP_2_ NRs also showed a robust photothermal stability, with an almost equal temperature elevation of 42.5 °C occurring in 5 cycles of laser irradiation (Figure 1j). Besides, the UV–vis absorbance spectrum and images of MoP_2_ NRs almost did not vary before/after five laser on/off cycles (Figure S7, Supporting Information), indicating the excellent photothermal stability of MoP_2_ NRs. The high photothermal conversion and photothermal stability highlight the great potential of MoP_2_ NRs in photothermal therapy.

### 2.2 Peroxidase Catalytic/Mild‐Photothermal Therapy Therapy In Vitro

**Figure 2:**
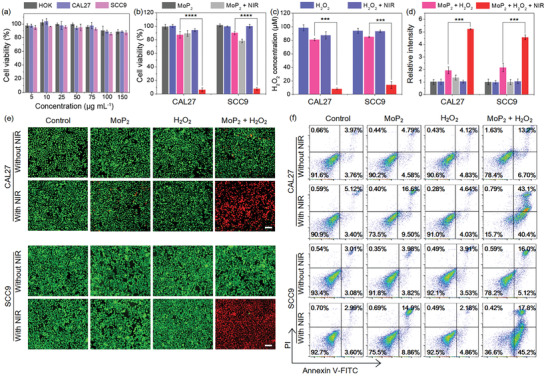
In vitro cytotoxicity assessment. a) Relative viabilities of HOK, CAL27, and SCC9 cells after incubation with different concentrations of MoP_2_ NRs for 24 h. b) The cell viability, c) H_2_O_2_, and d) •OH concentrations in CAL27 and SCC9 cells incubated with MoP_2_ NRs, H_2_O_2_, and MoP_2_ NRs + H_2_O_2_ with/without laser irradiation. e) Fluorescence images of the cells stained with calcein AM (live cells, green fluorescence) and PI (dead cells, red fluorescence) after different treatments (scale bar: 10 µm). f) Representative images of flow cytometry analysis of apoptotic/necrotic cells. Bar graphs show the mean ± SD. ***p < 0.001, and ****p < 0.0001.

Prior to using MoP_2_ NRs for biomedical applications, it is essential to assess their potential cytotoxicity. The cell viability of MoP_2_ NRs was investigated using a cell counting kit‐8 (CCK‐8) assay in three different cell lines, including one normal cell line (HOK) and two cancer cell lines (CAL27, SCC9). After 12 h incubation, MoP_2_ NRs were efficiently internalized by tumor cells (Figure S8, Supporting Information) showing favorable biocompatibility. As shown in **Figure** **2a**, all types of cells were treated with MoP_2_ NRs over a wide concentration range from 5 to 150 µg mL^–1^, no distinguishing cytotoxicity could be observed. Even at the high concentration (150 µg mL^–1^), more than 90% cells were still viable, verifying the excellent cytocompatibility of MoP_2_ NRs. Afterward, the antitumor performance of MoP_2_ NRs was investigated. As shown in Figure 2b, the cells treated with MoP_2_ NRs/H_2_O_2_/NIR alone, or combined treatment with MoP_2_ NRs + H_2_O_2_ and H_2_O_2_ + NIR, respectively, showed no distinguishable antitumor effect at the corresponding concentrations. Interestingly, the combination of MoP_2_ NRs + H_2_O_2_ with NIR irradiation significantly enhanced the antitumor efficacy. For example, nearly 93.7% of CAL27 cells and 92.3% of SCC9 cells were killed after treatment with MoP_2_ NRs (40 µg mL^–1^) + H_2_O_2_ (100 µmol mL^–1^) + NIR irradiation at the power density of 0.5 W cm^−2^ and the temperature was maintained at 43 °C for 10 min. Generally, the mild‐temperature hyperthermia (43 °C for 10 min) is insufficient to photodamage cancer cells. Therefore, in order to reveal the underlying mechanisms, the concentration of H_2_O_2_ in tumor microenvironment was detected by a commercial assay kit (Figure S9, Supporting Information).^[^ ^54^, ^55^ ^]^ Compared to the H_2_O_2_ or NIR irradiation alone treatment groups, the H_2_O_2_ levels in tumor microenvironment were significantly decreased, which might be attributed to the laser‐excited peroxidase‐like catalytic reaction of MoP_2_ NRs since abundant •OH radicals were generated as the sustain decreased H_2_O_2_ in the MoP_2_ NRs + H_2_O_2_ + NIR irradiation group (Figure 2c). To further explore this hypothesis, the laser‐excited catalytic activity of the MoP_2_ NRs was verified by oxidation of terephthalic acid.^[^ ^42^, ^45^, ^56^ ^]^ As shown in Figure 2d, the relative fluorescence intensity sharply increased in MoP_2_ NRs + H_2_O_2_ + NIR irradiation group, showing much higher concentration of •OH than that of the other groups. Similarly, the intracellular ROS level visualized by DCFH‐DA further confirmed that a prominent •OH generation in the MoP_2_ NRs + H_2_O_2_ + NIR irradiation group (Figures S10 and S11, Supporting Information). It is well‐known that •OH is a kind of ROS and generally has high reactivity due to the presence of unpaired valence shell electrons. Thus, the high level of •OH can cause irreversible lesions to tumor cells by destroying cellular biomolecule substances including lipids, proteins, and DNA.^[^ ^57^ ^]^

To visualize and confirm the improved therapeutic effect, the live/dead cells were distinguished by calcein‐acetoxymethyl (calcein‐AM, live cells, green fluorescence) and propidium iodide (PI, dead cells, red fluorescence) co‐staining after treatments. As shown in Figure 2e, most of cells were alive (green fluorescence) after treatments, whereas large amount of apoptosis/dead (red fluorescence) cells could be clearly observed in MoP_2_ NRs + H_2_O_2_ + NIR irradiation group, implying only the laser‐mediated MoP_2_ NRs peroxidase catalytic/mild‐photothermal therapy could effectively cause tumor cell apoptosis/dead. To further quantify cell apoptosis ratio, the treated cells were assessed by annexin V/PI co‐staining assay. As shown by the flow cytometry data in Figure 2f and Figure S12, Supporting Information, cell apoptosis obviously occurred in MoP_2_ NRs + H_2_O_2_ + NIR irradiation group, with total apoptosis ratios of CAL27 and SCC9 cells were 83.5% and 63.0%, respectively. These results were consistent with the CCK‐8 results and those in live/dead observation. Therefore, our preliminary data collectively evidenced that MoP_2_ NRs may be potential candidates as biocompatible and robust therapeutic agents for oral cancer therapy.

### 2.3 Antibacterial Activity In Vitro

**Figure 3:**
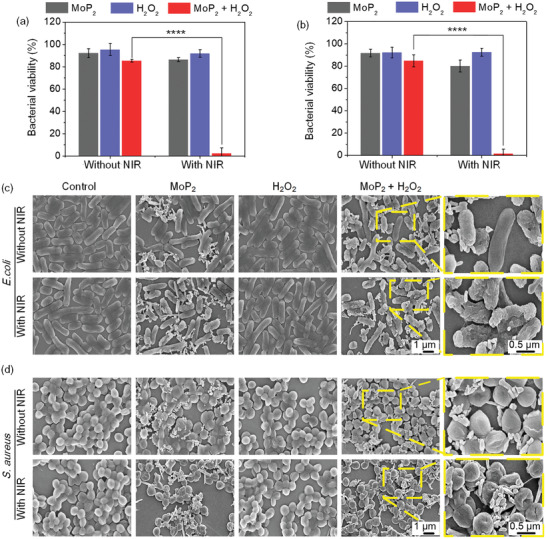
Antibacterial activity evaluation. Antibacterial efficacy on a) *E. coli* and b) *S. aureus* after treatments with PBS, MoP_2_ NRs (40 µg mL^–1^), H_2_O_2_ (100 µmol mL^–1^), and MoP_2_ + H_2_O_2_ without or with 808 nm irradiation for 10 min, respectively. SEM images of c) *E. coli* and d) *S. aureus* with different treatments (the regions with dashed‐yellow boarders are shown at higher magnification). Bar graphs show the mean ± SD. ****p < 0.0001.

In clinical cases, oral cavity associated wound infections caused by bacteria is one of the most prevalent complications during the treatment periods, which may cause systemic comorbidities, delay wound healing, and even increase the morbidity and mortality.^[^ ^58^ ^]^ Therefore, to address this challenge, the antibacterial activity of MoP_2_ NRs was evaluated both in Gram‐negative bacteria *E. coli* and Gram‐positive bacteria *S. aureus*. First, the bacteria viability was qualitatively evaluated by bacterial colony counting (**Figure** **3a**,b and Figure S13, Supporting Information), the statistical results revealed that MoP_2_ NRs incubation alone (40 µg mL^–1^), MoP_2_ + H_2_O_2_, or MoP_2_ + NIR (≈43 °C) were insufficient to eradicate bacteria, as more than 85.7% of *E. coli* and 80.3% of *S. aureus* were alive after 3 h incubation or combined with NIR irradiation. In contrast, nearly all of the bacteria were inactivated by treating with MoP_2_ + H_2_O_2_ + NIR, the bacterial viabilities were sharply reduced to 5.79 ± 5.02%, 3.01 ± 3.93% for *E. coli* and *S. aureus*, respectively, implying that NIR irradiation could synergistically promote the catalysis of H_2_O_2_, even at a low concentration (100 µmol L^–1^) of H_2_O_2_. The ROS level of bacteria in different experimental groups was detected by a reactive oxygen species assay kit. As shown in Figure S14, Supporting Information, no significant fluorescent was observed between MoP_2_ and H_2_O_2_ groups, indicating the low ROS level of bacteria. After treatment with MoP_2_ + H_2_O_2_, some fluorescence can be detected, implying few ROS were generated. In contrast, obvious green fluorescent increase was detected from MoP_2_ + H_2_O_2_ + laser groups, revealing ROS burst in the bacteria. The efficient decomposition of H_2_O_2_ generated a large amount of •OH, which subsequently oxidized the unsaturated fatty acids of membrane lipids and the amino acids of intracellular proteins,^[^ ^59^ ^]^ causing cellular membrane depolarization and irreparable DNA damage. Conversely, when the bacteria were treated with H_2_O_2_ alone or treated with NIR laser irradiation, negligible antibacterial effect was observed. Meanwhile, the bacterial viability was visualized by a Live/Dead BacLight kit, wherein live bacteria were stained green with SYTO 9 and the dead ones were stained red with propidium iodide (PI). As shown in Figure S15, Supporting Information, the intense red fluorescence from *E. coli* and *S. aureus* in the MoP_2_ NRs + H_2_O_2_ + laser irradiation groups revealed that bacteria become more sensitive and sterilized with mild‐PTT (≈43 °C).

SEM analysis of the morphological changes of bacteria with different treatments strongly confirmed that bacterial membrane and structure were significantly disrupted. As depicted in Figure 3c,d, the untreated *E. coli* and *S. aureus* showed typical rod‐like and spherical morphologies, respectively. Compared with the smooth surfaces of untreated bacteria, the surfaces of bacteria treated with MoP_2_, H_2_O_2_, or MoP_2_ + H_2_O_2_ maintained an intact membrane structure, indicating these treatments are insufficient to complete eradication of bacteria. In contrast, the distorted morphologies and wrinkled membrane structures of bacteria were obviously observed in MoP_2_ + H_2_O_2_ + NIR group (Figure S16, Supporting Information), where the high level of •OH induced severe cell damage by enhancing oxidative cleavage of bacterial components (proteins, oligosaccharides and DNA).^[^ ^60^ ^]^ Meanwhile, the membrane integrity of bacteria was disrupted, leading to cytoplasmic leakage (as the presented regions with dashed‐yellow boarders). Therefore, these encouraging results are evidencing that 808 nm laser‐potentiated peroxidase catalytic/mild‐photothermal therapy of MoP_2_ NRs not only improves the therapeutic efficiency of CDT, but also is showing potent antibacterial activity against pathogens.

### 2.4 In Vivo Antitumor Therapy and Toxicity Assessment

**Figure 4:**
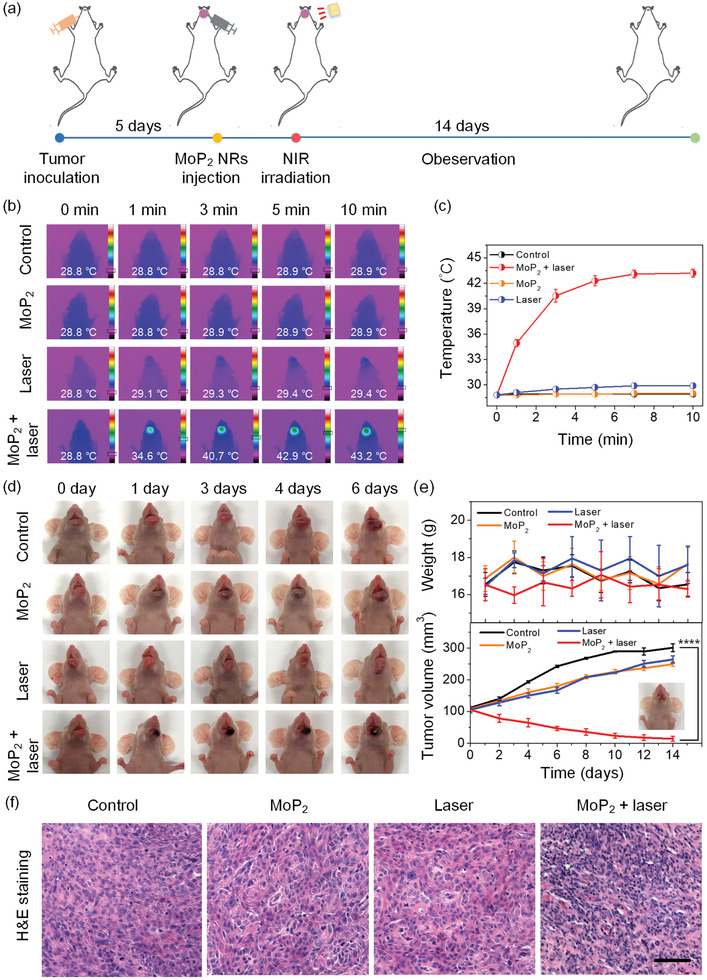
In vivo anti‐cancer therapy. a) A timeline with animation illustrating the treatment schedule. b) Infrared thermographic maps with color bar referring to the relative temperature and c) time‐dependent temperature increase curves in CAL27 tumor‐bearing mice irradiated by a 808 nm laser after separate intratumoral injection with 20 µL of PBS (the control, laser only groups) and 40 µg mL^–1^ MoP_2_ NRs (the MoP_2_ only, MoP_2_ + laser groups). d) Photographs of tumor volume variation of the four groups at 6 days post‐irradiation. e) Time‐dependent body‐weight curves and tumor growth curves of mice bearing orthotopic of CAL27 oral tumor in each group. (The inset shows the representative photo of tumor bearing mice at day 14 post MoP_2_ + laser treatment). f) H&E staining of tumor tissues after various treatments (scale bar: 50 µm). Bar graphs show the mean ± SD. ****p < 0.0001.

Considering the outstanding antitumor and antibacterial performance of laser‐potentiated peroxidase catalytic/mild‐photothermal therapy of MoP_2_ NRs in vitro, the therapeutic efficacy was further investigated in a CAL27 xenograft oral tumor model on BALB/c nude mice. When the tumors reached approximately 100 mm^3^ after injection of CAL27 cancer cells, the tumor‐bearing mice were randomly divided into four groups: I) Control (PBS), II) MoP_2_ NRs only, III) laser irradiation only, IV) MoP_2_ NRs + laser irradiation. As shown in the timeline with animation of **Figure** **4a**, after intratumor injection of 20 µL MoP_2_ NRs (5 mg kg^–1^),^[^ ^61^ ^]^ group (III) and (IV) were subsequently irradiated with an 808 nm laser for 10 min and the temperature changes of tumor sites were monitored by using an infrared thermal imaging camera simultaneously. As shown by the thermographic images (Figure 4b) and the corresponding time‐dependent temperature increase curves (Figure 4c), the temperature of tumors increased rapidly and was maintained at ≈43 °C in the MoP_2_ NRs + laser irradiation group, thereby avoiding the high temperature caused collateral damage to the healthy tissues (Figure S17, Supporting Information). In contrast, the temperatures of other groups were increased only slightly. Despite mild photothermal heating (43 °C) was observed in MoP_2_ NRs + laser irradiation group, remarkable tumor ablation was occurring with the prolonging of time. As shown in Figure 4d,e, the tumors volumes of the mice receiving MoP_2_ NRs or laser irradiation alone showed a negligible tumor suppression effect, enlarging nearly by 2.5‐fold in tumor volume at the 6 day after treatments. Remarkably, the tumors of the mice receiving MoP_2_ NRs + laser irradiation were significantly inhibited. At 14 days post‐irradiation, all of the tumors in MoP_2_ NRs + laser irradiation group were complete ablated without recurrence (Figures S18 and S19, Supporting Information), which may be attributed to the endogenous H_2_O_2_ of tumor sites efficiently converted into •OH via laser‐potentiated peroxidase catalytic reaction. As shown in Figure S20, Supporting Information, the relative red fluorescence intensity sharply increased in MoP_2_ NRs + NIR irradiation group, evidencing much higher level of ROS than that of the other groups. Combined with the NIR photothermal effect, the therapeutic efficacy was significantly improved compared to MoP_2_ NRs or laser irradiation treatment alone. These tendencies were consistent with the results of therapeutic assays in vitro. In addition, during two weeks treatments, no obvious sign of abnormal mouse behavior and body weight drop were noticed for all groups of mice, implying the excellent biocompatibility of the treatments. Afterward, the mice were anesthetized and sacrificed, the tumor tissues and major organs were sliced and stained by hematoxylin and eosin (H&E) staining. The histology analysis indicated that the tumor tissue sections under treatment with MoP_2_ NRs + laser irradiation exhibited serious cell damages, including irregular cell shrinkage, nuclear condensation, and tumor extracellular matrix decomposition. However, in MoP_2_ NRs or laser irradiation treatment groups, the histological sections showed infiltrating tumor cells with typical morphology and nuclear structures, demonstrating limited effects to restrain the tumor development (Figure 4f). In toxicity investigation, the major organs such as heart, liver, spleen, lung, and kidney were stained with H&E staining (Figure S21, Supporting Information). No pathological organ damage or inflammatory lesion could be observed, confirming the absence of evident toxic side effects. The in vivo toxicology was further investigated by haematological and biochemical analyses. As shown in Figure S22, Supporting Information, at the dosage of 8 mg kg^−1^ MoP_2_ NRs intravenous injection, no significant changes in blood hematology and biochemistry parameters, indicating that MoP_2_ NRs did not cause obvious toxicity or inflammation in vivo.

### 2.5 Terminal Deoxynucleotidyl Transferase dUTP Nick End Labeling and Immunohistochemistry Assays of Tumor Therapy

**Figure 5:**
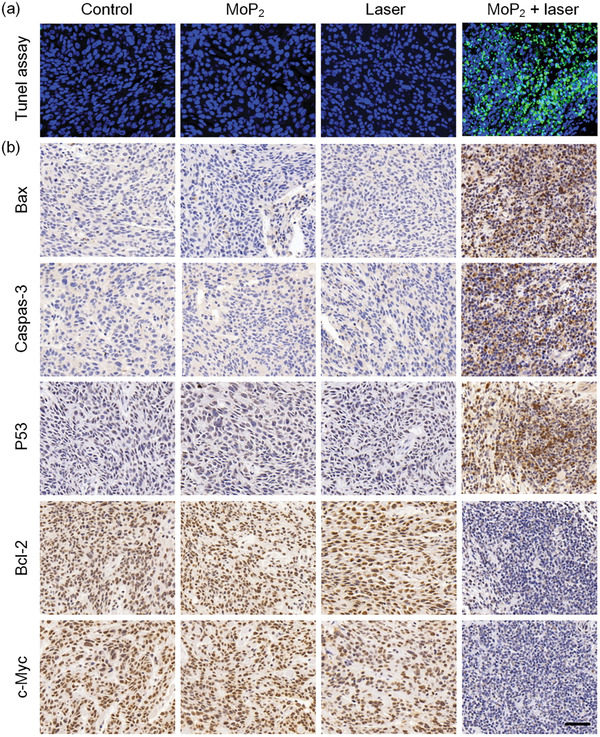
Immunofluorescence and immunohistochemical staining to observe the apoptosis of the tumor tissues. a) TUNEL assay and b) Bax, Caspases‐3, P53, Bcl‐2, and c‐Myc immunohistochemical staining of tumor tissues after various treatments (scale bar: 50 µm).

To further explore the underlying therapeutic mechanisms, cell apoptosis detection of the tumor sites was first detected by TUNEL (terminal deoxynucleotidyl transferase dUTP nick end labeling) staining, which is generally utilized to detect the DNA fragmentation resulting from apoptotic signaling cascades. As shown in **Figure** **5a**, no or few TUNEL‐positive cells (the TUNEL‐positive area is stained with green and normal cell nuclei is stained with blue) were observed from the phosphate buffered saline (PBS), MoP_2_ NRs, or laser irradiation groups. Instead, the tumors treated with MoP_2_ NRs + laser irradiation displayed more intense green fluorescence signals than any other group, implying significant cell apoptosis and also confirming the predominant treatment outcomes of MoP_2_ NRs + laser irradiation. To determine the potential apoptosis signal pathway, the pro‐apoptotic proteins including Bax, Caspases‐3, and P53 were detected by immunohistochemistry (IHC) analysis. The representative micrographs of IHC shown in Figure 5b indicated the up‐regulation of Bax (one pro‐apoptotic member of Bcl‐2 protein family) in the MoP_2_ NRs + laser irradiation group. Since Bax is usually triggered by pro‐apoptotic BH3‐only members (Bid, Bim, or PUMA),^[^ ^62^ ^]^ the mitochondrial apoptosis pathway maybe primarily responsible for inducing the apoptotic process. Moreover, Bax causes mitochondrial membrane permeabilization and release of cytochrome C, which would subsequently activate the downstream executioner Caspase‐3 to induce cell apoptosis. Indeed, the high expression of Caspase‐3 (a member of the cysteine‐aspartic acid protease family) was also clearly observed in MoP_2_ NRs + laser irradiation group, while amounts of Caspase‐3‐positive cells significantly increased after 14 days treatment, indicating the activation of mitochondrial apoptosis pathway. Previous studies have demonstrated that the production of ROS can facilitate the post‐translational modification of P53, which promotes the stability and activation of P53 protein.^[^ ^63^ ^]^ Considering that up‐regulation of P53 plays a crucial role for tumor suppression, including P53‐mediated cell cycle arrest and apoptosis,^[^ ^64^, ^65^ ^]^ in our study, the P53 protein expression was largely increased by •OH generated during the MoP_2_ NRs + laser irradiation treatment, Thereby, our results clearly revealed that the laser‐potentiated peroxidase catalytic reaction of MoP_2_ NRs effectively promoted the decomposition of tumor endogenous H_2_O_2_ into •OH. The high level of •OH subsequently disrupted the mitochondrial permeability transition pores, resulting in the loss of mitochondrial membrane potential and causing mitochondrial matrix osmotic swelling. As a result, the pro‐apoptotic proteins such as Bax, Caspases‐3, and P53 were significantly up‐regulated. Concomitantly with the down‐regulation of anti‐apoptotic modulators, including Bcl‐2 and c‐Myc, we can conclude that •OH induced mitochondrial apoptosis pathway is the dominant mechanism for highly efficient tumor eradication.

### 2.6 Biodistribution and Clearance

**Figure 6:**
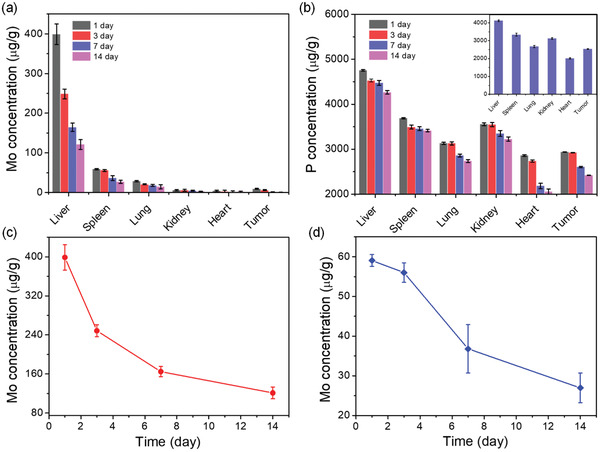
In vivo biodistribution and clearance of MoP_2_ NRs. a) Residual concentrations of Mo and b) P in different organs at different time points of 1, 3, 7, and 14 days post‐injection; (insert graph: The amount of P in a healthy mouse). c) The residual amount of Mo in the liver and d) spleen plotted versus time after intravenous injection of MoP_2_ NRs.

To assess the biodegradability of MoP_2_ NRs, we determined the biodistribution and clearance of MoP_2_ NRs in vivo. The MoP_2_ NRs solution (5 mg kg^−1^) was intravenously injected into CAL27 tumor‐bearing Balb/c mice (25 mice, n = 5). After 1, 3, 7 and 14 days post‐injection, the mice were scarified and the main organs including the heart, liver, spleen, lung, kidney, and tumor were collected and analyzed by inductively coupled plasma‐atomic emission spectrometry (ICP‐AES) to determine the biodistributions of Mo and P elements. As shown in **Figure** **6**, the Mo and P were mainly accumulated in the liver and spleen, which is probably due to the reticuloendothelial systems absorption of mice. At 1 d post‐injection, the Mo concentrations are as high as 398.82 µg g^−1^ (liver), 59.07 µg g^−1^ (spleen), 28.53 µg g^−1^ (lung), 5.93 µg g^−1^ (kidney), and 4.00 µg g^−1^ (heart), respectively. Meanwhile, MoP_2_ NRs were able to passive accumulate in tumors via the EPR effect, thereby making them suitable for tumor therapy. After 14 days injection, the residual amount of Mo was sharply decreased to 121.13 ± 12.173 µg g^−1^ (from 398.82 ± 25.861 µg g^−1^ at day 1) in liver and 26.96 ± 3.757µg g^−1^ in spleen (from 59.07 ± 1.511 µg g^−1^ at day 1), respectively. These results provide direct evidences that MoP_2_ NRs are biodegradable and can be excreted via the renal or fecal route in a period of 14 days.

## 3 Conclusion

In summary, our study demonstrates that a promising therapeutic strategy with efficient tumor CDT and anti‐infection capabilities was successfully developed. As‐prepared MoP_2_ NRs demonstrated strong optical absorption in NIR region and simultaneous favorable biocompatibility, showing great potential for the light‐mediated oral cancer therapy. When irradiated with 808 nm laser, MoP_2_ NRs could act as photosensitizers to efficiently capture the photo‐excited band electrons and valance band holes, exhibiting an enhanced peroxidase‐like catalytic activity for decomposition of tumor endogenous H_2_O_2_ to •OH. The high level of •OH subsequently broke the cellular biomolecules both in tumor cells and bacteria, facilitating cancer cells apoptosis and anti‐infective protection to achieve superior therapeutic efficacy. The corresponding experiments in vitro and in vivo confirmed the laser‐potentiated synergistic peroxidase catalytic/mild‐photothermal therapy that generated abundant •OH both in tumor cell and bacteria, thereby displaying notable inhibitory effects. Moreover, the anti‐cancer therapeutic mechanism investigation suggested that •OH induced mitochondrial apoptosis may be a dominant death signaling pathway to fight against oral cancer cells. Therefore, our work presented an innovative and multifunctional therapeutic modality via laser‐potentiated peroxidase catalytic/mild‐photothermal therapy, showing unique advantages in oral cancer therapy.

## 4 Experimental Section

### Materials

Molybdenum (99.9%, powder), red phosphorus (99.99%, lump), iodine (99.8%, particle), NMP were purchased from Aladdin Reagents. Dulbecco's modified Eagle's medium (DMEM), fetal bovine serum (FBS), trypsin‐EDTA, PBS (pH 7.4) were purchased from Gibco Life Technologies (AG, Switzerland). CCK‐8, Annexin V‐FITC/PI Apoptosis detection kit and hydrogen peroxide assay kit were obtained from Beyotime Biotech Co., Ltd. (Shanghai, China). Live/dead detection kit was obtained from Yeasen Biotech Co., Ltd. (Shanghai, China). Luria‐Bertan (LB) broth and LB agar were supplied by Thermo Fisher Scientific Inc. (Waltham, MA). The LIVE/DEAD BacLight Bacterial Viability Kit was obtained from Invitrogen (Carlsbad, CA). Fluorescein (FITC) Tunel Cell Apoptosis Detection Kit was obtained from Servicebio Biological Technology Co., Ltd. (Wuhan, China). The primary antibody against human Caspase‐3 was purchased from Boster Biological Technology Co., Ltd. (Wuhan, China), anti‐Bax antibody (bsm‐33283M) was purchased from Biosynthesis Biotechnology Inc. (Beijing, China), anti‐P53 antibody (GB11029), anti‐Bcl‐2 antibody (GB12008), anti‐c‐Myc antibody (GB13076) were purchased from Servicebio Biological Technology Co., Ltd. (Wuhan, China). All other chemical reagents were analytical grade and used without further purification. Ultrapure water (18.25 MU cm^−1^) was used in the experiments.

### Synthesis of Molybdenum Diphosphide Nanorods

MoP_2_ crystals were grown directly from the interaction between molybdenum and red phosphorus in a sealed evacuated quartz tube via high temperature reaction route. Briefly, Mo powders (100 mg) and excess red phosphorus lumps (300 mg) were placed into one end of the quartz tube and vacuum‐sealed under a pressure less than 2 × 10^−4^ Torr. Then, the quartz tubes were horizontally placed in a muffle furnace with the raw materials mixture located at the hot end and heating them at 850 °C for 18 h, followed by cooling to 30 °C at a rate of ≈2 °C min^−1^. Finally, MoP_2_ crystals were formed at the hot end of the quartz tube while the excess red phosphorus was visible at the cold end. The MoP_2_ NRs were subsequently prepared by ultrasonicating MoP_2_ crystals in NMP for 10 h. After sonication, the dispersion was centrifuged for 10 min at 4000 rpm. Then, the supernatant containing MoP_2_ NRs was collected by pipette and further centrifuged for 15 min at 10 000 rpm. The obtained precipitate was rinsed twice with ethanol to remove the residual NMP and re‐suspended in the aqueous solution.

### Characterization

The morphology and crystal structure of the as‐fabricated MoP_2_ NRs were characterized using Zeiss Supra 55 SEM (Carl Zeiss, Germany) and Tecnai G2 F20 S‐TWIN TEM (FEI, USA). The chemical composition of MoP_2_ NRs was determined by energy dispersive X‐ray spectroscopy, which was taken on the SEM (Oxford INCA 300). The crystal phase and purity were analyzed by XRD analyses and performed on a SmartLab X‐ray diffractometer (Rigaku, Japan) with Cu Ka irradiation (*λ* = 1.5406 Å). The chemical composition and purity of the obtained products were analyzed by XPS and carried out using the Thermo Fisher ESCALAB 250Xi XPS (Thermo Fisher, USA). The amounts of MoP_2_ in solution were quantified by ICP‐AES (IRIS Intrepid II XSP, thermo Electron Corporation, USA). The UV–vis–NIR absorption spectra were recorded on the TU‐1810 ultraviolet–visible spectrophotometer (Purkinje General Instrument Co. Ltd. Beijing, China).

### Photothermal‐Conversion Property of Molybdenum Diphosphide Nanorods

To quantitatively determine the photothermal conversion efficiency of MoP_2_ NRs, 1 mL aqueous dispersions of the MoP_2_ NRs with different concentrations were respectively maintained in a 1 cm path length quartz cuvette and irradiated by a semiconductor diode laser (808 nm, KS‐810F‐8000, Kai Site Electronic Technology Co., Ltd. Shaanxi, China) at a power density of 1.0 W cm^−2^ for 10 min. The thermographs were recorded by an infrared thermal imaging camera (Ti27, Fluke) at certain time intervals (30 s). Temperature elevation of same concentration (40 µg mL^–1^) of the nanoparticles with an 808 nm laser at different power densities (0.2, 0.4, 0.6, 0.8, and 1.0 W cm^−2^) for 10 min was also recorded. The photostability of MoP_2_ NRs was examined by means of five on/off laser cycles. To be specific, the temperature changes of nanoparticles solution (40 µg mL^–1^) were tracked during the irradiation under a NIR laser (808 nm, 1 W cm^–2^) for 10 min, then the laser was switched off until spontaneously reduced to room temperature.

### Cell Culture and Cellular Toxicity Assay

CAL27 and SCC9 cells (human OSCC cell line) were acquired from Wuhan University (Wuhan, China). HOK cells (Human oral keratinocyte) were obtained from the cell bank of the Chinese Academy of Sciences (Shanghai, China). CAL27 cells, SCC9 cells and HOK cells were cultured in DMEM medium supplied with 10% FBS in a humidified atmosphere at 37 °C with 5% CO_2_, respectively. The cell toxicity of MoP_2_ NRs was evaluated by a standard CCK‐8 assay. First, CAL27 cells, SCC9 cells and HOK cells were plated on the 48‐well plates (2 × 10^4^ cells per well) with 400 µL of complete medium and incubated for 24 h at 37 °C with 5% CO_2_, respectively. Then, the medium was replaced with 200 µL of newly complete medium containing various concentrations of MoP_2_ NRs and cultured for another 24 h. Afterward, the culture medium was removed, and the cells were washed with PBS for three times and then 10 µL CCK‐8 per well was added and further cultured for 1.5 h at 37 °C. Subsequently, the relative cell viability was quantitatively determined by the optical density (OD) at 450 nm as an indicator of viable cells obtained on a microplate spectrophotometer (Varioskan Flash 4.00.53, Finland). The cell growth inhibition was calculated according to the following formula: Cell viability (%) = (OD_test_ − OD_blank_)/(OD_control_ − OD_blank_) × 100%.

### In Vitro Peroxidase Catalytic/Mild‐Photothermal Therapy Therapy against Tumor Cells

To investigate the MoP_2_ NRs based peroxidase catalytic/mild‐PTT performance in vitro, CAL27 and SCC9 cells (2 × 10^4^ cells per well) were seeded into 48‐well plates and cultured for overnight. Then, the cell was sorted into control, MoP_2_, H_2_O_2_, MoP_2_ (40 µg mL^–1^) + H_2_O_2_ (100 µmol mL^–1^) groups with or without NIR irradiation. After the supernatant was removed, a 200 µL of newly medium mixed with 40 µg mL^–1^ of MoP_2_ NRs was added into the MoP_2_, MoP_2_ + H_2_O_2_, MoP_2_ + NIR, MoP_2_ + H_2_O_2_ + NIR groups, and 100 µmol mL^−1^ of H_2_O_2_ was respectively added into the H_2_O_2_, MoP_2_ + H_2_O_2_, H_2_O_2_ + NIR, MoP_2_ + H_2_O_2_ + NIR groups and maintained for 12 h. Subsequently, four NIR groups were exposed to the laser at 0.5 W cm^–2^ for 10 min and the relative cell viability was assessed by the CCK‐8, while the temperature was detected by thermal imager during the experiment. In addition, to visualize and confirm the improved therapeutic effect after different treatments, each group of cells was stained with a live/dead detection kit (Yeasen Biotech, shanghai, China) for 15 min to distinguish the live cells and the dead cells (live cells were stained green with Calcein AM and dead ones were stained red with PI). Fluorescent images were captured using an inverted fluorescence microscope (IX71, Olympus, Germany). To further assess the in vitro peroxidase catalytic/mild‐PTT efficacy of MoP_2_ NRs, CAL27, and SCC9 cells were respectively plated into 24‐well plates (5 × 10^4^ cells per well) and incubated for 24 h. Afterward, the treatment was the same as descripted in above experiment. Finally, the cells were collected, stained with the Annexin V‐FITC Apoptosis Kit (Beyotime Biotech, Shanghai, China), and detected by flow cytometry (BD FACSCelesta, New Discovery, Shanghai, China).

### Detection of Hydrogen Peroxide

CAL27 cells and SCC9 cells (1 × 10^4^ cells per well) were seeded into 96‐well plates and incubated for overnight. Next, the cells’ treatment was in accordance with the previously described experiment. After incubation with MoP_2_ NRs and exposure on NIR irradiation (0.5 W cm^–2^), the H_2_O_2_ content of the H_2_O_2_, MoP_2_ + H_2_O_2_, H_2_O_2_ + NIR, MoP_2_ + H_2_O_2_ + NIR groups was respectively measured using a hydrogen peroxide assay kit (Beyotime Biotech, Shanghai, China) according to the manufacturer's protocol. Briefly, the culture media and cells digested by Trypsin of each group were gathered to determine the H_2_O_2_ content. Finally, 50 µL of each sample and 100 µL of reagent were mixed at room temperature for 30 min, and then the absorbance at 560 nm was measured using a microplate reader (Multisken sky, ThermoFisher, China). The H_2_O_2_ concentration (µmol mL^–1^) was interpolated from the standard curve made from known concentration solutions (Figure S9, Supporting Information).

### Detection of Hydroxyl Radical (•OH)

The catalytic activity of the MoP_2_ NRs with low concentration of H_2_O_2_ (100 µmol mL^–1^) is detected by the terephthalic acid (TA) fluorescence method. It has been reported that terephthalic acid (TA) can react with •OH to generate a highly fluorescent product, 2‐hydroxyterephthalic acid (TAOH).^[^ ^54^ ^]^ After incubation with MoP_2_ NRs and irradiation by 808 nm laser for 10 min, the culture media and cells digested by Trypsin of each group were collected to mixed with TA (500 µmol mL^–1^). The solutions were gently shaken in an orbital incubator at 37 °C for 12 h in the dark then changes of fluorescence emission peak at 435 nm were measured by a fluorescence spectroscopy (F‐4600, HITACHI, Japan). The enhanced percentage was calculated as (F_test_ − F_blank_)/(F_control_ − F_blank_), where F_blank_ is the intial quantity of fluorescence intensity that absence of TA.

### Antibacterial Assay

The *E. coli* (ATCC 25922) and *S. aureus* (ATCC 43300) were cultured and resuspended in 10 mL of LB broth, respectively, and grown in a 37 °C shaker overnight with 200 rpm rotation. Afterward, 100 µL of each bacterial suspension was transferred to 10 mL of fresh LB broth for subculture and harvested at the exponential growth phase. Subsequently, 500 µL of each bacterial suspension was serially diluted 10 times to maintain the final concentration of bacteria at 1 × 10^5^–1 × 10^6^ CFU mL^−1^. The *E. coli* or *S. aureus* bacteria were respectively added into eight groups: I) Bacteria, II) bacteria + MoP_2_, III) bacteria + H_2_O_2_, IV) bacteria + MoP_2_ + H_2_O_2_, V) bacteria + NIR, VI) bacteria + MoP_2_ + NIR, VII) bacteria + H_2_O_2_ + NIR, and VIII) bacteria + MoP_2_ + H_2_O_2_ + NIR. Afterward, 200 µL of diluted bacteria suspensions were respectively transferred to 1.8 mL fresh LB broth. The final concentrations of MoP_2_ NRs, H_2_O_2_, and bacteria were 40 µg mL^–1^, 100 µmol L^–1^, and 1.0 × 10^5^ CFU mL^–1^, respectively. For NIR groups, the mixtures were irradiated with an 808 nm laser for 10 min at a certain power density, an infrared thermal imager was applied to record the temperature changes. After incubation for 3 h, the bacteria were diluted with sterile PBS. 100 µL of the bacterial suspensions were spread on LB agar plates and cultured at 37 °C for 16 h, respectively. The antibacterial efficacy was determined by the standard plate‐based counting method. The bacteria viability was also assessed by a Live/Dead BacLight Kit: the bacterial suspension was mixed with 2 µL of SYTO 9 and 2 µL of propidium iodide for 15 min incubation in the dark at room temperature. The bacterial solution was centrifuged at 3000 rpm for 4 min and visualized and photographed under a fluorescence microscope.

### Morphology Observation of Bacteria

After different treatments, the morphological changes of the *E. coli* and *S. aureus* bacteria were monitored by SEM. Each sample of the bacterial suspensions: I) PBS, II) MoP_2_ (40 µg mL^–1^), III) H_2_O_2_ (100 µmol mL^–1^), IV) MoP_2_ + H_2_O_2_, V) PBS + NIR, VI) MoP_2_ + NIR, VII) H_2_O_2_ + NIR, VIII) MoP_2_ + H_2_O_2_ + NIR were centrifuged at 3000 rpm for 5 min and washed with PBS three times. Then, the bacterial precipitation was dispersed in 2.5% glutaraldehyde solution and fixed at 4 °C for overnight. The fixed bacteria were washed with PBS three times, dehydrated by sequential ethanol series (25, 50, 80, and 100 wt%, respectively, for 10 min), and dry completely at room temperature. Afterward, the bacteria were sputter‐coated with gold (30 s, 30 mA), and observed by SEM (Zeiss Sigma 300).

### Animals and Tumor Models

The female BALB/c nude mice (4–6 weeks old) were purchased from Hunan Slac Laboratory Animal Co., Ltd. (Hunan, China). All research experiments were performed in accordance with the regulations formulated by the Animal Care and Use Committee of the Shenzhen Institutes of Advanced Technology, Chinese Academy of Sciences (IACUC NO. SIAT‐IACUC‐190605‐CLS‐LZB‐A0752). The orthotopic CAL27 oral cancer tumor was implanted in lower lip by injecting 1 × 10^6^ CAL27 cells in 20 µL of sterile PBS to cause tumor formation.

### In Vivo Peroxidase Catalytic/Mild‐Photothermal Therapy Therapy

When the tumors reached ≈100 mm^3^, the CAL27 tumor‐bearing mice were randomly divided into four groups: Control group, MoP_2_ NRs‐only group, laser‐only group, and MoP_2_ + laser group. The MoP_2_ NRs were administered in a intratumoral injection. In details, the latter two groups were injected with 20 µL of MoP_2_ NRs (5 mg kg^–1^) into the center of tumor, while the former two groups were only injected with 20 µL of PBS. After post‐injection, the laser irradiation groups were irradiated with the 808 nm NIR laser at the power density of 0.5 W cm^–2^ for 10 min, the temperature of the tumors and infrared thermographic maps were monitored by an infrared thermal imaging camera (Ti27, Fluke, USA). The tumor volumes and body weights of the mice were recorded every two days and the tumor volumes was calculated as *V* = 1/2 (Length × Width^2^).

### Terminal Deoxynucleotidyl Transferase dUTP Nick End Labeling and Immunohistochemistry Assays

At day 14 after the treatment, the mice were euthanatized and the tumors were excised. The collected tumors were fixed in 4% paraformaldehyde for 24 h, embedded in paraffin, sectioned into 5 µm, stained with hematoxylin and eosin (H&E) to observe the morphology and status of cancer cells. For the TUNEL apoptosis staining, the sections were stained with the Fluorescein (FITC) Tunel Cell Apoptosis Detection Kit (Servicebio, Wuhan, China) according to the manufacturer's instructions. Moreover, immunohistochemical analysis (Bax, Caspases‐3, P53, Bcl‐2, and c‐Myc) was performed. The tumor sections were respectively incubated with anti‐Bax antibody (bsm‐33283M; 1:100; Bioss, Beijing, China), anti‐Caspase‐3 antibody (BA3592; 1:500; Boster, Wuhan, China), anti‐P53 antibody (GB11029; 1:500; Servicebio, Wuhan, China), anti‐Bcl‐2 antibody (GB12008; 1:500; Servicebio, Wuhan, China), anti‐c‐Myc antibody (GB13076; 1:100; Servicebio, Wuhan, China) overnight at 4 °C, and subsequent incubation with 100 µL of 1 × secondary antibody solution for 50 min (GB23303, 1:200, Servicebio, Wuhan, China). The signals were developed using the Pierce DAB Substrate Kit (34002, Thermo Fisher Scientific IL, USA) following the manufacture's protocol.

### In Vivo Biodistribution and Clearance

The MoP_2_ NRs solution (5 mg kg^−1^) was intravenously injected into CAL27 tumor‐bearing Balb/c mice (25 mice, *n* = 5). After 1, 3, 7, and 14 days post‐injection, the mice were euthanized and scarified. The main organs including the heart, liver, spleen, lung, kidney, and tumor were collected, wet‐weighed, and digested in 5 mL of 65% nitric acid. Finally, the Mo and P concentrations were measured by ICP‐AES.

### Statistical Analysis

All data were presented as means ± standard deviations (SD). Statistical comparisons was analyzed using one‐way analysis of variance (ANOVA) with Bonferroni multiple comparison test using SPSS software (SPSS Inc., USA). In all cases, a value of *p < 0.05 was considered to be statistically significant and that **p < 0.01, ***p < 0.001, or ****p < 0.0001 were considered to be highly significant.

## Conflict of Interest

The authors declare no conflict of interest.

## Supporting information

Supporting Information

## Data Availability

The data that support the findings of this study are available from the corresponding author upon reasonable request.

## References

[1] R. L. Siegel , K. D. Miller , A. Jemal , CA‐Cancer J. Clin. 2020, 70, 7. 31912902 10.3322/caac.21590

[2] A. A. Hussein , M. N. Helder , J. G. de Visscher , C. R. Leemans , B. J. Braakhuis , H. C. W. de Vet , T. Forouzanfar , Eur. J. Cancer 2017, 82, 115. 28654785 10.1016/j.ejca.2017.05.026

[3] J. Ferlay , I. Soerjomataram , R. Dikshit , S. Eser , C. Mathers , M. Rebelo , D. M. Parkin , D. Forman , F. Bray , Int. J. Cancer 2015, 136, E359. 25220842 10.1002/ijc.29210

[4] F. Inchingolo , L. Santacroce , A. Ballini , S. Topi , G. Dipalma , K. Haxhirexha , L. Bottalico , I. A. Charitos , Int. J. Environ. Res. Public Health 2020, 17, 3168. 10.3390/ijerph17093168 PMC7246763 32370133

[5] J. Robledo‐Sierra , D. P. Ben‐Amy , E. Varoni , R. Bavarian , J. L. Simonsen , B. J. Paster , W. G. Wade , A. R. Kerr , D. E. Peterson , E. F. Lau , Oral Dis. 2019, 25, 28. 31140694 10.1111/odi.13107

[6] S. L. Chuang , W. W. Y. Su , S. L. S. Chen , A. M. F. Yen , C. P. Wang , J. C. Y. Fann , S. Y. H. Chiu , Y. C. Lee , H. M. Chiu , D. C. Chang , Y. Y. Jou , C. Y. Wu , H. H. Chen , M. K. Chen , S. T. Chiou , Cancer 2017, 123, 1597. 28055109 10.1002/cncr.30517

[7] T. Morikawa , T. Shibahara , T. Nomura , A. Katakura , M. Takano , Cancers 2020, 12, 2771. 10.3390/cancers12102771 PMC7601016 32992486

[8] A. H. Alsarraf , O. Kujan , C. S. Farah , J. Oral Pathol. Med. 2018, 47, 104. 29130527 10.1111/jop.12660

[9] P. Szturz , J. B. Vermorken , Oral Oncol. 2020, 101, 104492. 31837576 10.1016/j.oraloncology.2019.104492

[10] D. K. Zanoni , P. H. Montero , J. C. Migliacci , J. P. Shah , R. J. Wong , I. Ganly , S. G. Patel , Oral Oncol. 2019, 90, 115. 30846169 10.1016/j.oraloncology.2019.02.001 PMC6417804

[11] S. H. Huang , E. Hahn , S. I. Chiosea , Z. Y. Xu , J. S. Li , L. Shen , B. O'Sullivan , Oral Oncol. 2020, 102, 104563. 31918174 10.1016/j.oraloncology.2019.104563

[12] A. Singh , M. Mair , H. Singhvi , A. Mahuvakar , D. Nair , S. Nair , P. Chaturvedi , Oral Oncol. 2019, 90, 8. 30846181 10.1016/j.oraloncology.2019.01.011

[13] J. L. Geiger , D. J. Adelstein , Oral Oncol. 2020, 102, 104584. 32032863 10.1016/j.oraloncology.2020.104584

[14] Y. Cho , H. I. Yoon , I. J. Lee , J. W. Kim , C. G. Lee , E. C. Choi , S. H. Kim , K. C. Keum , Head Neck 2019, 41, 3916. 31430016 10.1002/hed.25928

[15] E. Sjamsudin , T. Maulina , A. Cipta , A. Iskandarsyah , A. Hardianto , M. Nandini , A. Kasim , H. Y. Yusuf , Oral Maxillofac. Surg. 2018, 22, 83. 29332186 10.1007/s10006-018-0672-3

[16] Y. Lim , M. Totsika , M. Morrison , C. Punyadeera , Theranostics 2017, 7, 4313. 29158828 10.7150/thno.21804 PMC5695015

[17] M. F. Cheng , C. S. Lin , Y. H. Chen , P. J. Sung , S. R. Lin , Y. W. Tong , C. F. Weng , Mar. Drugs 2017, 15, 224. 10.3390/md15070224 PMC5532666 28714874

[18] P. Gholizadeh , H. Eslami , M. Yousefi , M. Asgharzadeh , M. Aghazadeh , H. S. Kafil , Biomed. Pharmacother. 2016, 84, 552. 27693964 10.1016/j.biopha.2016.09.082

[19] M. P. Veve , S. L. Davis , A. M. Williams , J. E. McKinnon , T. A. Ghanem , Oral Oncol. 2017, 74, 181. 28943204 10.1016/j.oraloncology.2017.09.011

[20] M. B. Gobic , A. Zubovic , R. Cerovic , A. Dekanic , D. Marzic , G. Zamolo , J. Craniomaxillofac. Surg. 2018, 46, 135. 29203089 10.1016/j.jcms.2017.11.003

[21] B. W. Yang , Y. Chen , J. L. Shi , Chem. Rev. 2019, 119, 4881. 30973011 10.1021/acs.chemrev.8b00626

[22] Z. M. Tang , Y. Y. Liu , M. Y. He , W. B. Bu , Angew. Chem., Int. Ed. 2019, 58, 946.

[23] Y. Liu , W. Y. Zhen , L. H. Jin , S. T. Zhang , G. Y. Sun , T. Q. Zhang , X. Xu , S. Y. Song , Y. H. Wang , J. H. Liu , ACS Nano 2018, 12, 4886. 29727164 10.1021/acsnano.8b01893

[24] Y. Yang , Z. Liu , Chem 2020, 6, 2127.

[25] Y. J. Wang , L. N. Shi , Z. F. Ye , K. S. Guan , L. L. Teng , J. H. Wu , X. Yin , G. S. Song , Nano Lett. 2020, 20, 176. 31777250 10.1021/acs.nanolett.9b03556

[26] X. W. Wang , X. Y. Zhong , Z. Liu , L. Cheng , Nano Today 2020, 35, 100946.

[27] L. S. Lin , J. B. Song , L. Song , K. M. Ke , Y. J. Liu , Z. J. Zhou , Z. Y. Shen , J. Li , Z. Yang , W. Tang , G. Niu , H. H. Yang , X. Y. Chen , Angew. Chem., Int. Ed. 2018, 57, 4902. 10.1002/anie.201712027 29488312

[28] Q. F. Chen , J. Zhou , Z. Chen , Q. Luo , J. Xu , G. B. Song , ACS Appl. Mater. Interfaces 2019, 11, 30551. 31397998 10.1021/acsami.9b09323

[29] C. Zhang , W. Bu , D. Ni , S. Zhang , Q. Li , Z. Yao , J. Zhang , H. Yao , Z. Wang , J. Shi , Angew. Chem., Int. Ed. 2016, 55, 2101. 10.1002/anie.201510031 26836344

[30] S. Gao , H. Lin , H. Zhang , H. Yao , Y. Chen , J. Shi , Adv. Sci. 2019, 6, 1801733. 10.1002/advs.201801733 PMC6364502 31168441

[31] Z. H. Zhao , W. Q. Wang , C. X. Li , Y. Q. Zhang , T. R. Yu , R. F. Wu , J. Y. Zhao , Z. Liu , J. Liu , H. J. Yu , Adv. Funct. Mater. 2019, 29, 1905013.

[32] J. Shan , X. Li , K. Yang , W. Xiu , Q. Wen , Y. Zhang , L. Yuwen , L. Weng , Z. Teng , L. Wang , ACS Nano 2019, 13, 13797. 31696705 10.1021/acsnano.9b03868

[33] M. Huo , L. Wang , H. Zhang , L. Zhang , Y. Chen , J. Shi , Small 2019, 15, 1901834. 10.1002/smll.201901834 31207096

[34] X. Wang , L. Fan , L. Cheng , Y. Sun , X. Wang , X. Zhong , Q. Shi , F. Gong , Y. Yang , Y. Ma , Z. Miao , Z. Zha , iScience 2020, 23, 101281. 32622263 10.1016/j.isci.2020.101281 PMC7334425

[35] B. Ma , S. Wang , F. Liu , S. Zhang , J. Duan , Z. Li , Y. Kong , Y. Sang , H. Liu , W. Bu , J. Am. Chem. Soc. 2018, 141, 849. 30541274 10.1021/jacs.8b08714

[36] Q. Chen , Y. Luo , W. X. Du , Z. Liu , S. J. Zhang , J. H. Yang , H. L. Yao , T. Z. Liu , M. Ma , H. R. Chen , ACS Appl. Mater. Interfaces 2019, 11, 18133. 31046230 10.1021/acsami.9b02905

[37] G. Liu , J. Zhu , H. Guo , A. Sun , P. Chen , L. Xi , W. Huang , X. Song , X. Dong , Angew. Chem., Int. Ed. 2019, 58, 18641. 10.1002/anie.201910815 31605417

[38] S. M. Dong , J. T. Xu , T. Jia , M. S. Xu , C. N. Zhong , G. X. Yang , J. R. Li , D. Yang , F. He , S. L. Gai , P. P. Yang , J. Lin , Chem. Sci. 2019, 10, 4259. 31057754 10.1039/c9sc00387h PMC6471739

[39] Z. H. Pu , L. S. Amiinu , M. Wang , Y. S. Yang , S. C. Mu , Nanoscale 2016, 8, 8500. 27065023 10.1039/c6nr00820h

[40] Y. N. Gao , M. L. Zhang , J. J. Ding , S. Hong , J. Masa , S. Z. Liu , Z. Y. Sun , Electrochem. Commun. 2018, 97, 27.

[41] T. L. Wu , S. J. Chen , D. Zhang , J. Hou , J. Mater. Chem. A 2015, 3, 10360.

[42] W. X. Zhu , C. Tang , D. N. Liu , J. L. Wang , A. M. Asiri , X. P. Sun , J. Mater. Chem. A 2016, 4, 7169.

[43] Y. Y. Gao , H. Y. Li , J. Y. Wang , J. Y. Ma , H. S. Ren , Nanomaterials 2019, 9, 1270.

[44] W. Y. Yin , J. Yu , F. T. Lv , L. Yan , L. R. Zheng , Z. J. Gu , Y. L. Zha , ACS Nano 2016, 10, 11000. 28024334 10.1021/acsnano.6b05810

[45] Z. Wang , Z. M. Liu , C. K. Su , B. W. Yang , X. X. Fei , Y. Li , Y. Q. Hou , H. N. Zhao , Y. X. Guo , Z. F. Zhuang , H. Q. Zhong , Z. Y. Guo , Curr. Med. Chem. 2019, 26, 1788. 28933294 10.2174/0929867324666170920152529

[46] M. Wen , J. H. Wang , R. F. Tong , D. N. Liu , H. Huang , Y. Yu , Z. K. Zhou , P. K. Chu , X. F. Yu , Adv. Sci. 2018, 6, 1801321. 10.1002/advs.201801321 PMC6325597 30643723

[47] Z. B. Li , G. H. Luo , W. P. Hu , J. L. Hua , S. Y. Geng , P. K. Chu , J. Zhang , H. Y. Wang , X. F. Yu , Angew. Chem., Int. Ed. 2020, 59, 20568. 10.1002/anie.202008379 32666703

[48] Y. Yang , W. J. Zhu , Z. L. Dong , Y. Chao , L. Xu , M. W. Chen , Z. Liu , Adv. Mater. 2017, 29, 1703588. 10.1002/adma.201703588 28833643

[49] C. Zhang , L. S. Y. Yong , X. Dong , X. Zhang , X. Liu , Z. Gu , Y. Zhao , Z. Hu , Adv. Healthcare Mater. 2016, 5, 2776. 10.1002/adhm.201600633 27717238

[50] J. G. Choi , L. T. Thompson , Appl. Surf. Sci. 1996, 93, 143.

[51] P. Xiao , M. A. Sk , L. Thia , X. Ge , R. J. Lim , J.‐Y. Wang , K. H. Lim , X. Wang , Energy Environ. Sci. 2014, 7, 2624.

[52] C. Deng , F. Ding , X. Y. Li , Y. F. Guo , W. Ni , H. Yan , K. N. Sun , Y. M. Yan , J. Mater. Chem. A 2016, 4, 59.

[53] H. H. Xie , Z. B. Li , Z. B. Sun , J. D. Shao , X. F. Yu , Z. N. Guo , J. H. Wang , Q. L. Xiao , H. Y. Wang , Q. Q. Wang , H. Zhang , P. K. Chu , Small 2016, 12, 4136. 27329254 10.1002/smll.201601050

[54] Y. X. Jin , Q. Yang , L. Liang , L. Ding , Y. X. Liang , D. D. Zhang , B. L. Wu , T. Yang , H. L. Liu , T. T. Huang , H. Shen , H. L. Tu , Y. B. Pan , Y. C. Wei , Y. Yang , F. L. Zhou , J. Exp. Clin. Cancer Res. 2018, 37, 277. 30454068 10.1186/s13046-018-0948-3 PMC6245615

[55] L. Wang , H. Gao , X. Y. Yang , X. C. Liang , Q. C. Tan , Z. R. Chen , C. Zhao , Z. Y. Gu , M. S. Yu , Y. Zheng , Y. F. Huang , L. Y. Zhu , T. J. C. Jacob , L. W. Wang , L. X. Chen , Clin. Exp. Pharmacol. Physiol. 2018, 45, 1019. 29884989 10.1111/1440-1681.12979

[56] H. J. Sun , N. Gao , K. Dong , J. S. Ren , X. G. Qu , ACS Nano 2014, 8, 6202. 24870970 10.1021/nn501640q

[57] S. S. Gao , H. Lin , H. X. Zhang , H. L. Yao , Y. Chen , J. L. Shi , Adv. Sci. 2019, 6, 1801733. 10.1002/advs.201801733 PMC6364502 31168441

[58] Z. Fan , B. Liu , J. Wang , S. Zhang , Q. Lin , P. Gong , L. Ma , S. Yang , Adv. Funct. Mater. 2014, 24, 3933.

[59] Z. Z. Wang , Y. Zhang , E. G. Ju , Z. Liu , F. F. Cao , Z. W. Chen , J. S. Ren , X. G. Qu , Nat. Commun. 2018, 9, 3334. 30127408 10.1038/s41467-018-05798-x PMC6102211

[60] L. Z. Gao , K. M. Giglio , J. L. Nelson , H. Sondermann , A. J. Travis , Nanoscale 2014, 6, 2588. 24468900 10.1039/c3nr05422e PMC3951791

[61] B. K. Wang , J. H. Wang , Q. Liu , H. Huang , M. Chen , K. Y. Li , C. Z. Li , X. F. Yu , P. K. Chu , Biomaterials 2014, 35, 1954. 24331707 10.1016/j.biomaterials.2013.11.066

[62] M. Pérez‐Hernández , P. D. Pino , S. G. Mitchell , M. Moros , G. Stepien , B. Pelaz , W. J. Parak , E. M. Gálvez , J. Pardo , J. M. de la Fuente , ACS Nano 2015, 9, 52. 25493329 10.1021/nn505468v

[63] S. J. Tseng , Z. X. Liao , S. H. Kao , Y. F. Zeng , K. Y. Huang , H. J. Li , C. L. Yang , Y. F. Deng , C. F. Huang , S. C. Yang , P. C. Yang , I. M. Kempson , Nat. Commun. 2015, 6, 6456. 25739372 10.1038/ncomms7456 PMC4366491

[64] S. H. Moon , C. H. Huang , S. L. Houlihan , K. K. Regunath , W. A. Freed‐Pastor , J. P. Morris IV , D. F. Tschaharganeh , E. R. Kastenhuber , A. M. Barsotti , R. Culp‐Hill , W. Xue , Y. J. Ho , T. Baslan , X. Li , A. Mayle , E. D. Stanchina , L. Zender , D. R. Tong , A. D'Alessandro , S. W. Lowe , C. Prives , Cell 2019, 176, 564. 30580964 10.1016/j.cell.2018.11.011 PMC6483089

[65] Y. H. Liu , X. X. Hu , C. C. Han , L. N. Wang , X. N. Zhang , X. M. He , X. B. Lu , BioEssays 2015, 37, 1277. 26445307 10.1002/bies.201500093 PMC8638220

