# Supplementary material for: Molybdenum Diphosphide Nanorods with Laser‐Potentiated Peroxidase Catalytic/Mild‐Photothermal Therapy of Oral Cancer
Source: Adv Sci (Weinh). 2021 Oct 31;9(1):2101527. doi: 10.1002/advs.202101527 (PMC8728868; doi:10.1002/advs.202101527)
Supplement: Supplementary file 1 — Supporting Information [file ADVS-9-2101527-s001.pdf]

© 2021 Wiley-VCH GmbH

ADVANCED SCIENCE
Open Access

# Supporting Information

for *Adv. Sci.*, DOI: 10.1002/advs.202101527

# Molybdenum Diphosphide Nanorods with Laser-Potentiated Peroxidase Catalytic/Mild-Photothermal Therapy of Oral Cancer

*Min Qian, Ziqiang Cheng, Guanghong Luo, Massimiliano Galluzzi, Yuehong Shen*, Zhibin Li*, Hongyu Yang,* and Xue-Feng Yu*

# Supporting Information

**Molybdenum Diphosphide Nanorods with Laser-Potentiated Peroxidase Catalytic/Mild-Photothermal Therapy of Oral Cancer**

*Min Qian,[#] Ziqiang Cheng,[#] Guanghong Luo, Massimiliano Galluzzi, Yuehong Shen*, Zhibin Li*, Hongyu Yang* and Xue-Feng Yu*

M. Qian, Dr. Y. Shen, Prof. H. Yang

Department of Oral and Maxillofacial Surgery, Guangdong Provincial High-level Clinical Key Specialty, Guangdong Province Engineering Research Center of Oral Disease Diagnosis and Treatment, Peking University Shenzhen Hospital, 518036, Guangdong, P. R. China

E-mail: yuehongshen@hotmail.com (Y. Shen); hyyang192@hotmail.com (H. Yang)

Dr. Z. Cheng, Dr. M. Galluzzi, Prof. Z. Li, Prof. Xue-Feng Yu

Materials and Interfaces Center, Shenzhen Institute of Advanced Technology, Chinese Academy of Sciences, Shenzhen 518055, Guangdong, P. R. China

E-mail: lzbszu@163.com (Z. Li)

G. Luo

Department of Radiation Oncology, The Second Clinical Medical College, Jinan University (Shenzhen People's Hospital), Shenzhen 518020, Guangdong, P. R. China

E-mail: lzbszu@163.com (Z. Li)

## Experimental Section/Methods

### Stability evaluation

The same amount of $MoP_2$ NRs (15 μg $mL^{-1}$) were dispersed in different solutions (water, PBS, DMEM, and DMEM supplemented with 10% FBS) and the photographs were acquired at regular intervals.

### Degradability evaluation

The $MoP_2$ NRs (15 μg $mL^{-1}$) were dispersed in the phosphate-buffered saline (PBS; pH = 7.2) and then incubated at a 37°C shaker for 7 days. The degradation behavior was observed by visual inspection and UV-vis-NIR absorption spectra.

### Calculation of the extinction coefficient

The calculation of $MoP_2$ NRs of the extinction coefficient. (α) The $MoP_2$ NRs aqueous solutions with different concentrations was measured with a ultraviolet spectrophotometer. The absorption intensity over the characteristic length of the cuvette (A/L) at 808 nm was normalized to the concentration (C)

measured by ICP-AES. According to the Lambert-Beer law: A/L = αC,[1] α presents the extinction coefficient, there is a linear trend observed from A/L versus C, and α of the $MoP_2$ NRs is calculated as 20.5 L $g^{-1}$ $cm^{-1}$.

**Calculation of the photothermal conversion efficiency of $MoP_2$ NRs**

The photothermal conversion efficiency ($\eta$) can be calculated by Eqs. (1-4) [2,3]

$\eta = (hS(T_{max}-T_{surr})-Q_{dis})/I(1-10^{-A})$ (1)

$hS = \sum mC_p/\tau_S$ (2)

$\tau_S = - t/\ln\vartheta$ (3)

$\vartheta = (T - T_{surr})/(T_{max} - T_{surr})$ (4)

where $h$ is the heat transfer coefficient, $S$ is the surface area of the container, $\tau_S$ is the time constant for heat transfer from the system, $m$ is mass of products ($m$ = 1 g), $C_p$ is specific heat capacity of solvent ($C_p$, water = 4.2 $Jg^{-1}°C^{-1}$), and $\tau_S$ = 139.6 s is obtained from the following Figure S5. $Q_{dis}$ is measured independently to be 365.4 mW, $hS$ is obtained from Eqs(2) ($hS$ = 1*4.2/139.6= 30.1 mW/°C), $T_{max}$ is the equilibrium temperature of $MoP_2$ NRs, $T_{surr}$ is the ambient temperature of the surroundings, $I$ is the laser power density (0.5 W/$cm^2$), and $A$ is the absorbance of $MoP_2$ NRs at 808 nm ($A_{808}$ = 0.512). Accordingly, $\eta$ = {[30.1*(42.7-28.4)-365.4]/[500*(1-$10^{-0.512}$)]}*100%= 18.8%.

**Cellular uptake**

CAL27 cells and SCC9 cells were plated on a 48-well plate (2 × $10^4$ cells per well) for 24 h and exposure to $MoP_2$ NRs for another 12 h. Then, the cells were rinsed with PBS for three times and the micrographs of oral cancer cells containing $MoP_2$ NRs were taken by a microscope (IX71, Olympus).

**Intracellular ROS measurement**

The intracellular ROS level was evaluated by fluorescence imaging. The oral cancer cells (CAL27 and SCC9) were cultured in 48-well plate with fresh medium for overnight, respectively. After different treatments, the culture medium was removed and the cells were washed thrice with PBS and stained with DCFH-DA probe (10 μM) for 20 min at 37°C. The cells were visualized by an inverted fluorescent microscope (DCFH-DA: λex = 488 nm, λem = 525 nm).

**Tumor ROS detection by DHE assay**

The tumor tissues were dissected and embedded in optimal cutting temperature medium and cut into 8 μm cryosections. After washing with PBS, sections were incubated with dihydroethidium (DHE) solution (1:500, D7008, SIGMA) at 37 °C for 30 min. Cell nuclei were stained with DAPI (G0002; Servicebio, Wuhan, China). Five randomly selected fields from each section were imaged with a fluorescence microscope (Nikon Eclipse, Japan).

# Results

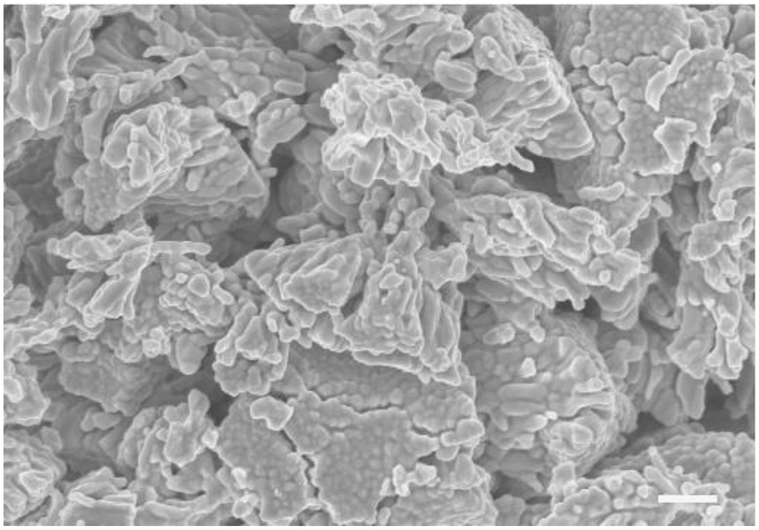

**Figure S1.** SEM image of $MoP_2$ powder before the ultrasonication-assisted exfoliation process (scale bar: 1 μm).

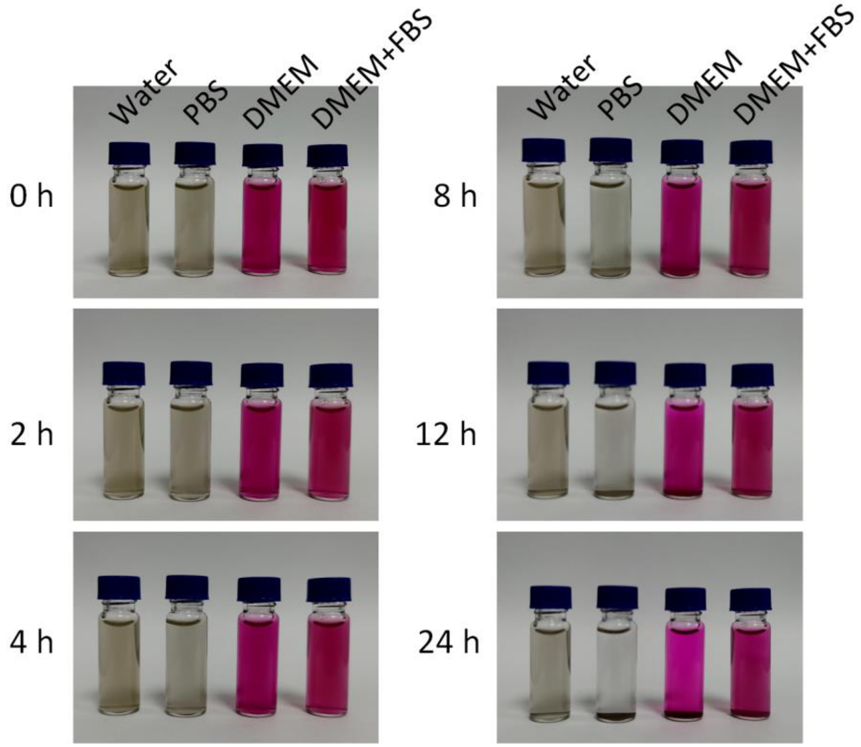


**Figure S2.** Digital images of $MoP_2$ NRs dispersed in a variety of media (water, PBS, DMEM, and DMEM supplemented with 10% FBS) with different periods of time.

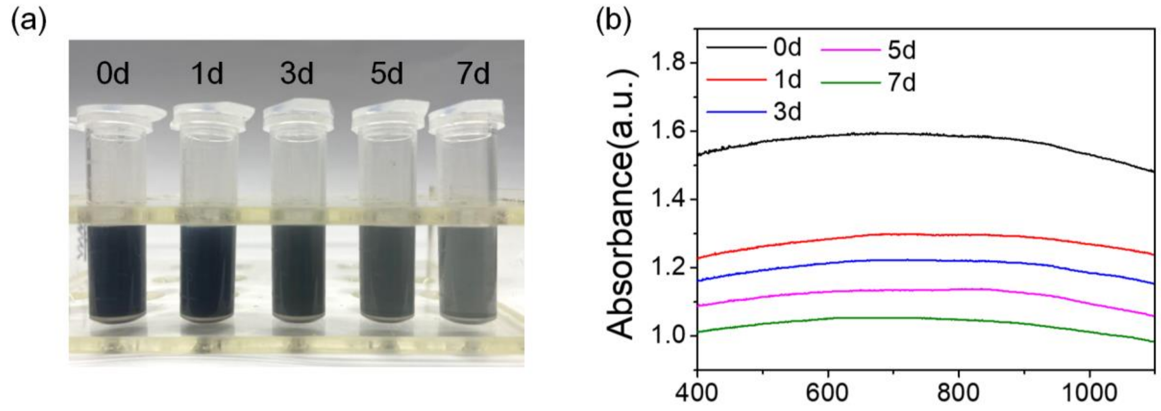


**Figure S3**. Degradation behavior of $MoP_2$. (a) Photographs of $MoP_2$ dispersed in PBS at predetermined time intervals. (b) UV-vis spectra of $MoP_2$ PBS dispersions at different degradation time points.

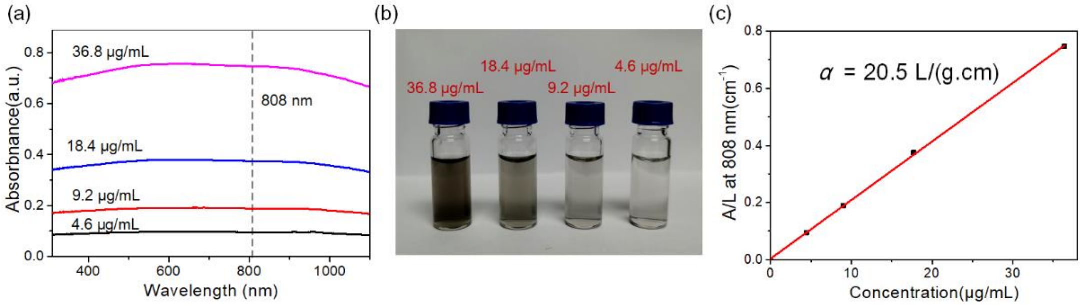


**Figure S4.** The optical detection of $MoP_2$ NRs. (a) The ultraviolet-visible (UV-Vis)-NIR absorbance and (b) digital images of $MoP_2$ NRs with different concentrations. (c) Concentration-dependent absorbance of $MoP_2$ NRs at 808 nm.

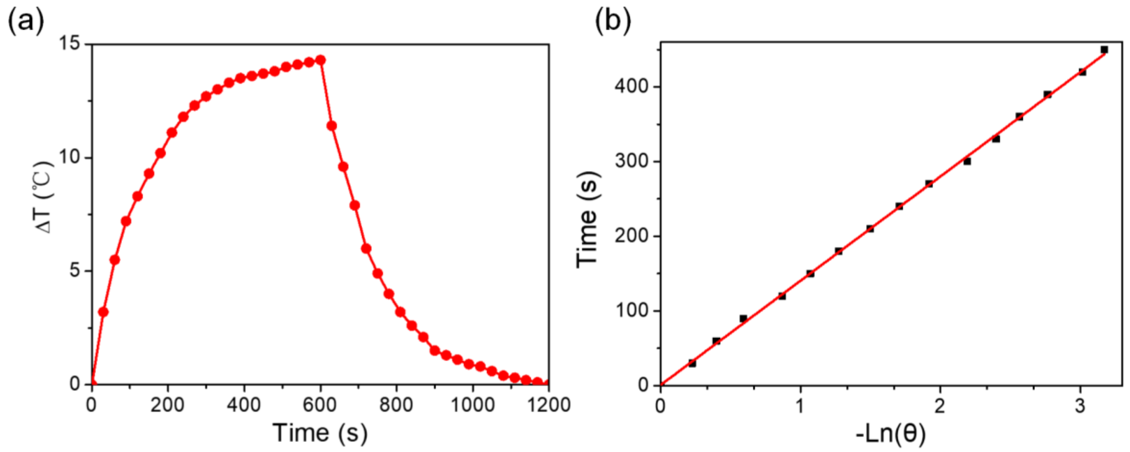


**Figure S5**. Photothermal conversion efficiency of $MoP_2$ NRs. (a) Heating and cooling curves of $MoP_2$ NRs solution irradiated with 808 nm NIR laser for 10 min and then naturally cooled to room temperature, (b) plot of cooling time versus the negative natural logarithm of the driving force temperature ($\tau_s$ = 139.6 s).

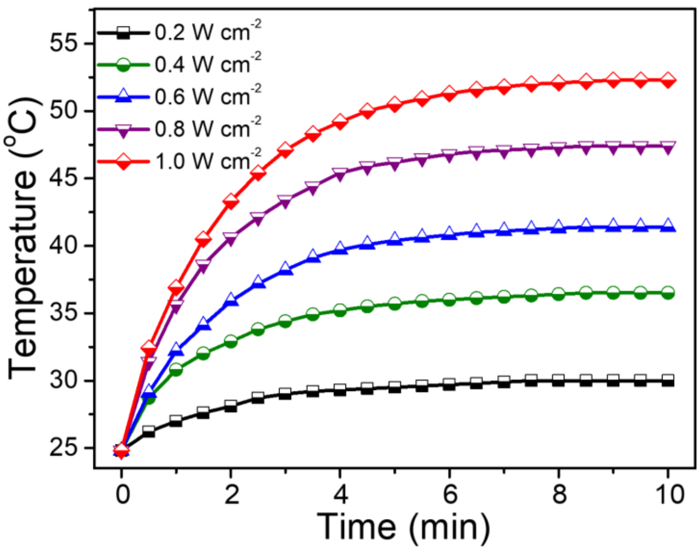


**Figure S6.** Photothermal heating curves of $MoP_2$ NRs solutions (40 μg $mL^{-1}$) irradiated by an 808 nm laser with different power densities (0.2, 0.4, 0.6, 0.8, 1.0 W $cm^{-2}$).

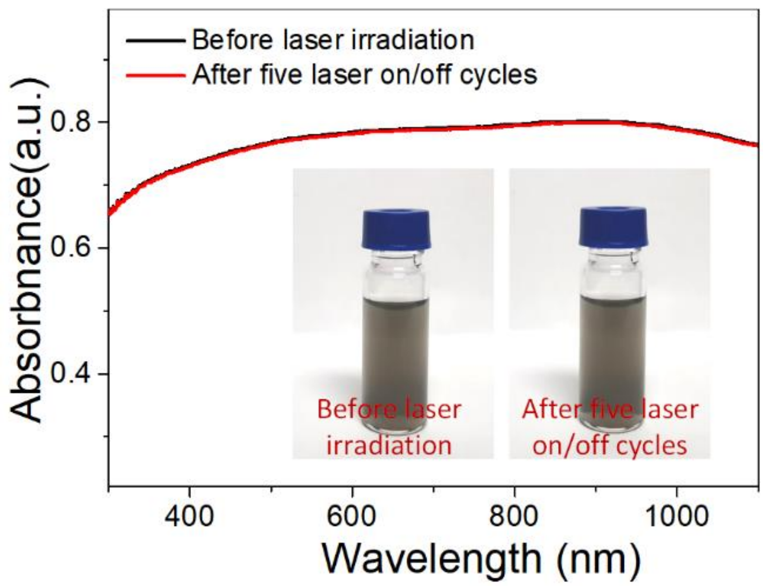


**Figure S7.** The UV-Vis-NIR absorbance and digital images of $MoP_2$ NRs before/after five laser on/off cycles.

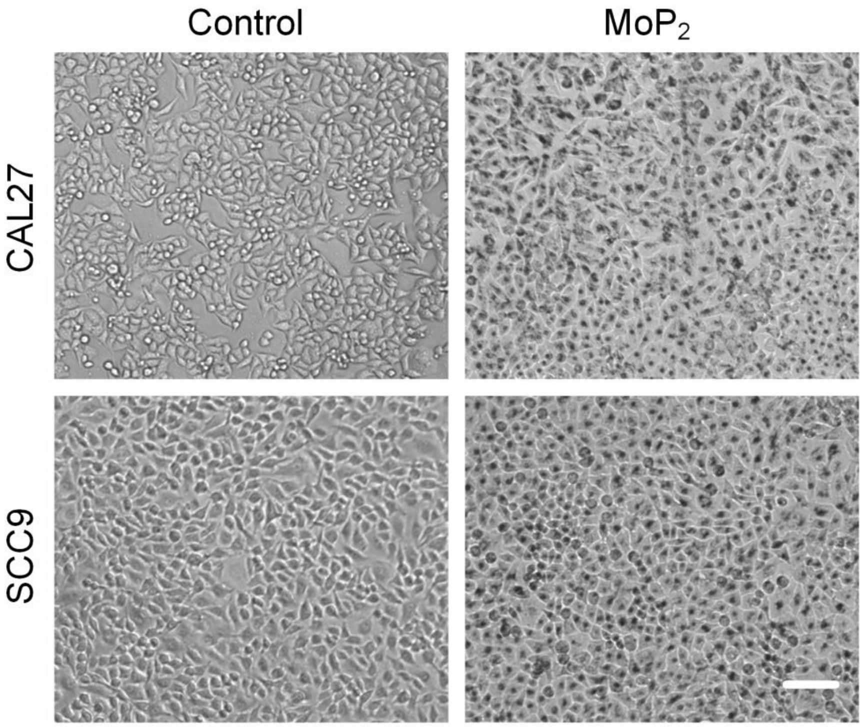


**Figure S8.** Cellular uptake of $MoP_2$ NRs in oral cancer cells: Bright-field images of oral cancer cells before and after uptake of $MoP_2$ NRs (scale bars: 10 µm for all panels).

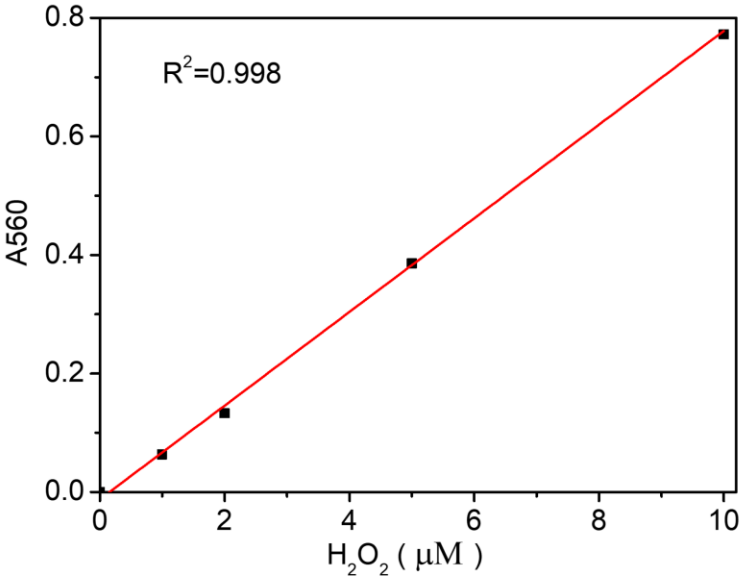


**Figure S9.** The $H_2O_2$ standard curve acquired from a series of $H_2O_2$ standard solutions.

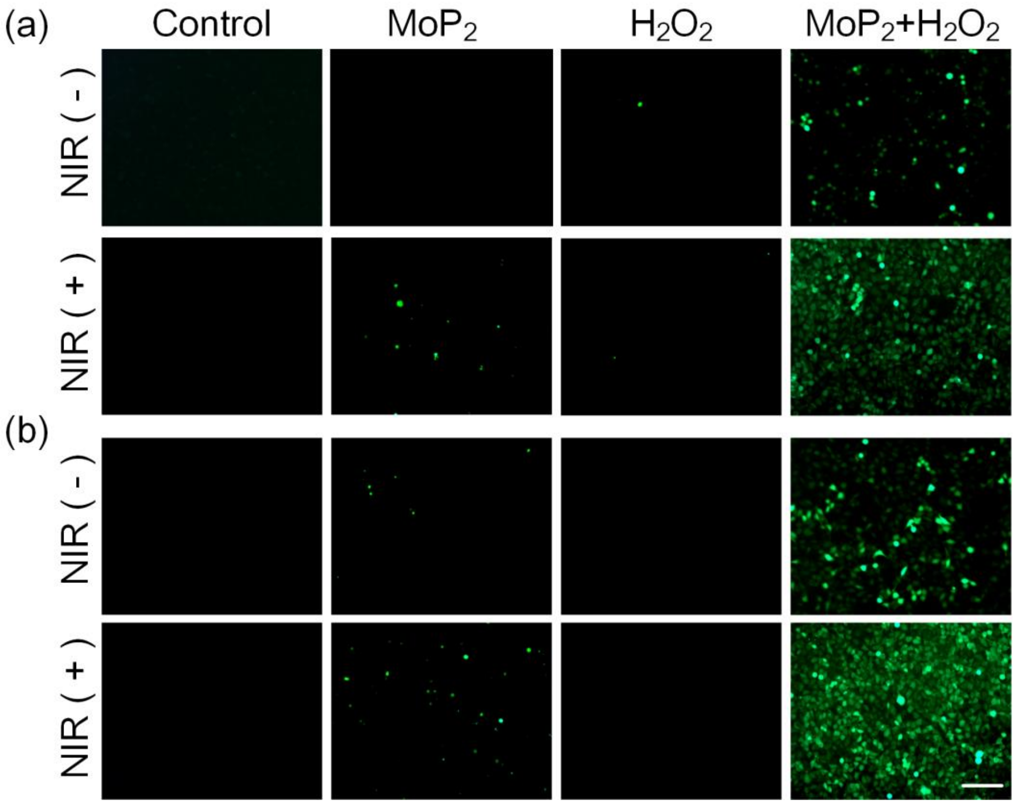


**Figure S10.** Fluorescent images of ROS generation in (a) CAL27 and (b) SCC9 oral cancer cells treated with different groups (Green fluorescence indicates ROS; scale bars: 10 μm).

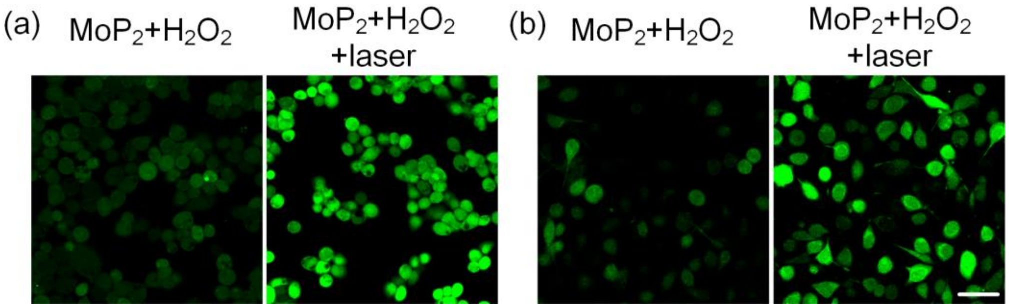


**Figure S11**. Confocal fluorescent images of ROS generation in (a) CAL27 and (b) SCC9 oral cancer cells treated with $MoP_2$ + $H_2O_2$ and $MoP_2$ + $H_2O_2$ + laser, respectively (scale bars: 50 μm).

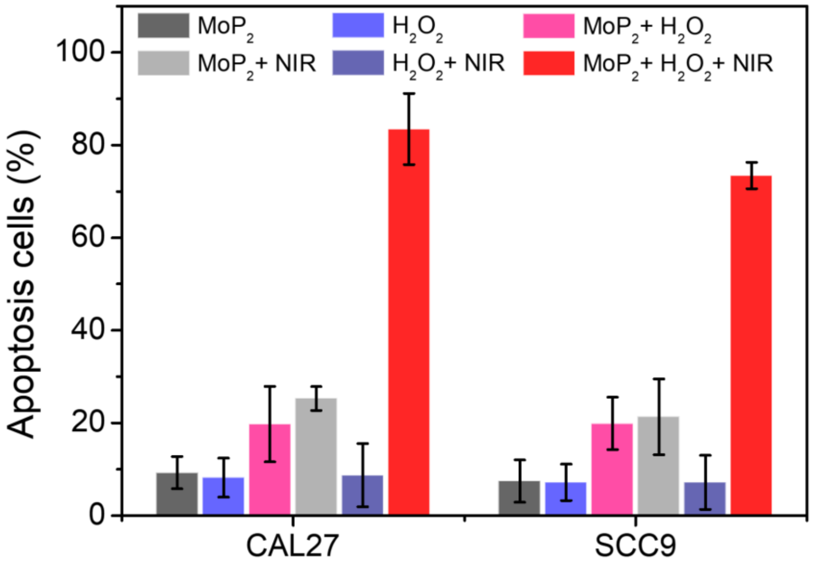


**Figure S12.** The total apoptotic rate of CAL27 and SCC9 oral cancer cells after various treatments.

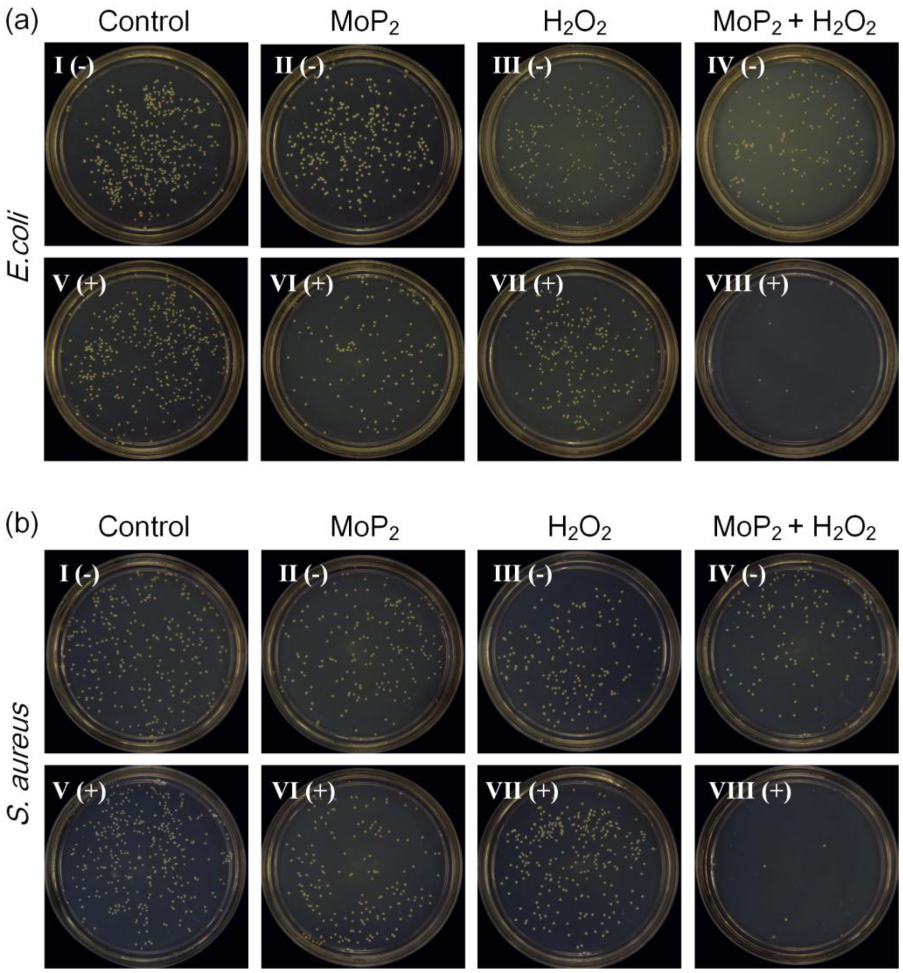


**Figure S13.** Photographs of bacterial colonies formed by (a) *E. coli* and (b) *S. aureus* after treatment with (I) PBS, (II) $MoP_2$, (III) $H_2O_2$, (IV) $MoP_2$ + $H_2O_2$, (V) PBS + NIR, (VI) $MoP_2$ + NIR, (VII) $H_2O_2$ + NIR and (VIII) $MoP_2$ + $H_2O_2$ + NIR. Concentration: $MoP_2$ 40 μg $mL^{-1}$, $H_2O_2$ 100 μmol $mL^{-1}$.

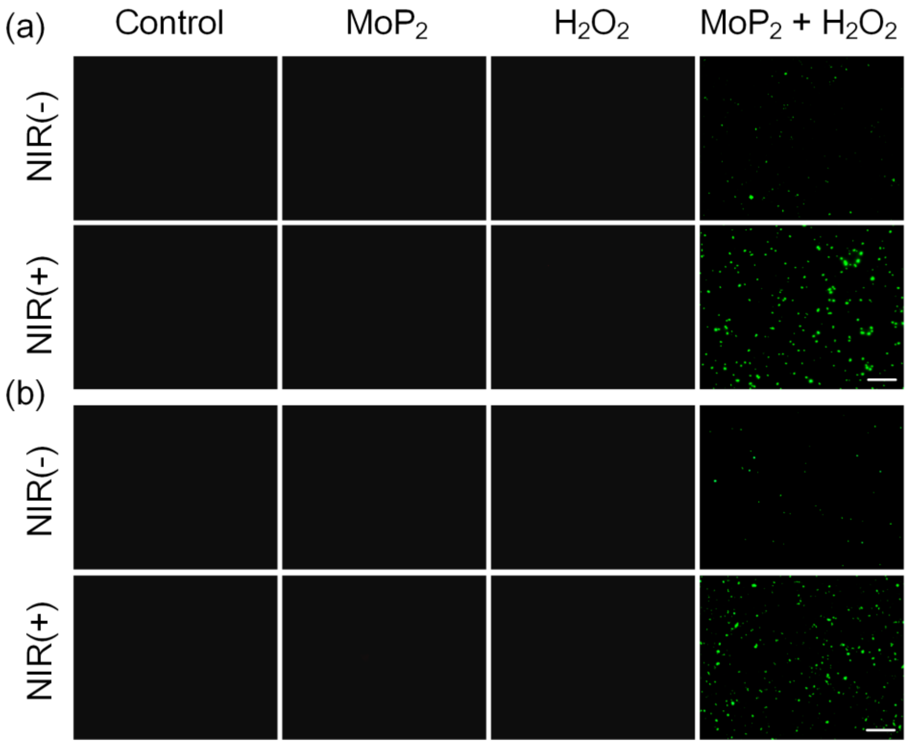


**Figure S14**. ROS fluorescence staining of (a) *E. coli*, (b) *S. aureus* after incubating with $MoP_2$ NRs, $H_2O_2$, and $MoP_2$ NRs + $H_2O_2$ with/without laser irradiation (scale bar: 50 μm).

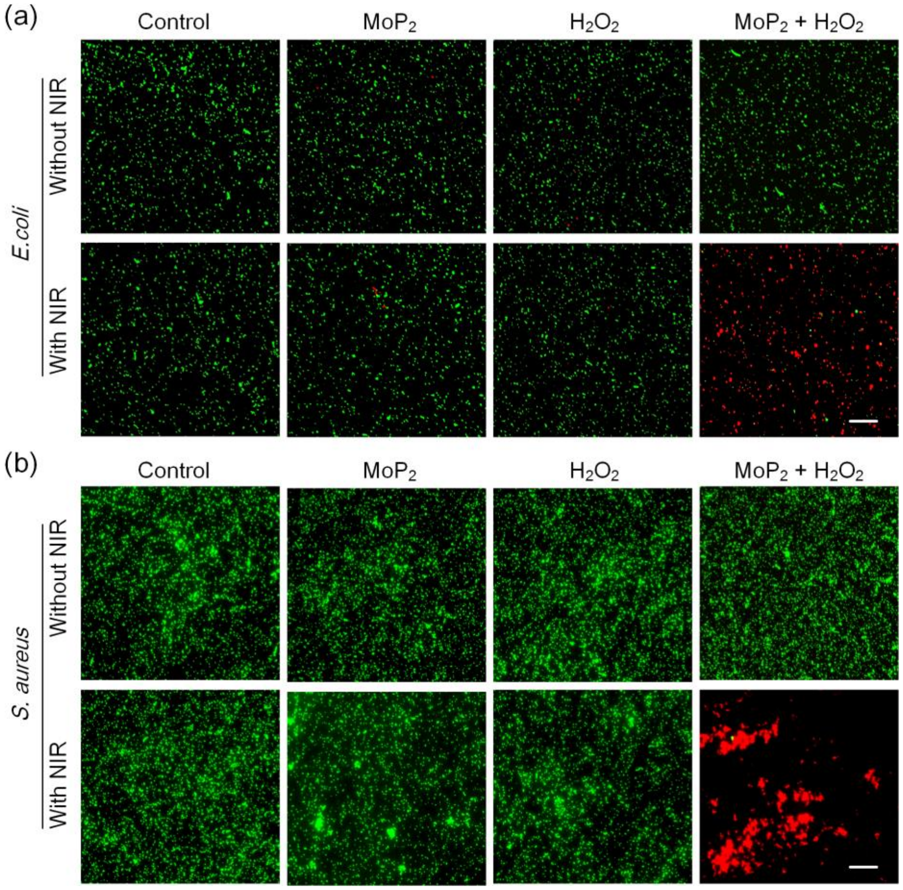


**Figure S15.** Fluorescent images of the bacteria in different groups with Live/Dead staining (viable bacteria are stained green fluorescence with SYTO 9 and dead ones are stained red fluorescence with PI). Scale bar: 50 μm for all panels.

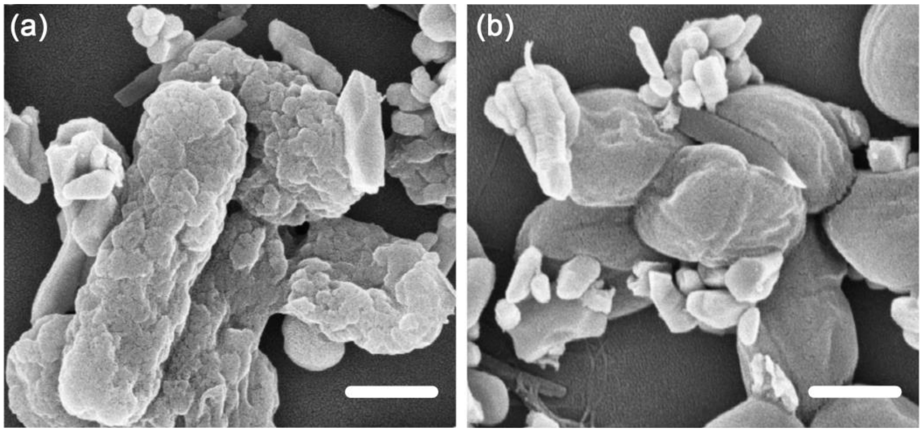


**Figure S16**. High-resolution SEM images of (a) *E.coli* and (b) *S aureus* incubated with $MoP_2$ NRs (scale bar: 500 nm).

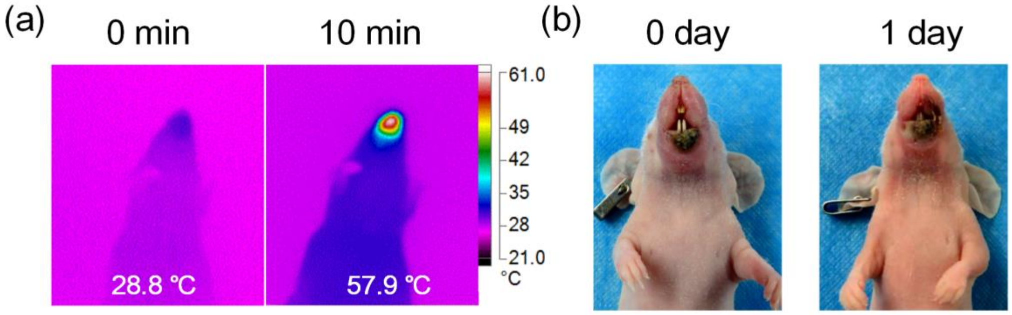


**Figure S17** *In vivo* photothermal cancer therapy. (a) Infrared thermographic maps and (b) photographs of tumor ablation in the CAL27 tumor-bearing nude mouse irradiated by the 808nm laser (1 $Wcm^{-2}$) for 10 min after intratumoral injection with 20 μL of $MoP_2$ NRs (5 mg $kg^{-1}$).

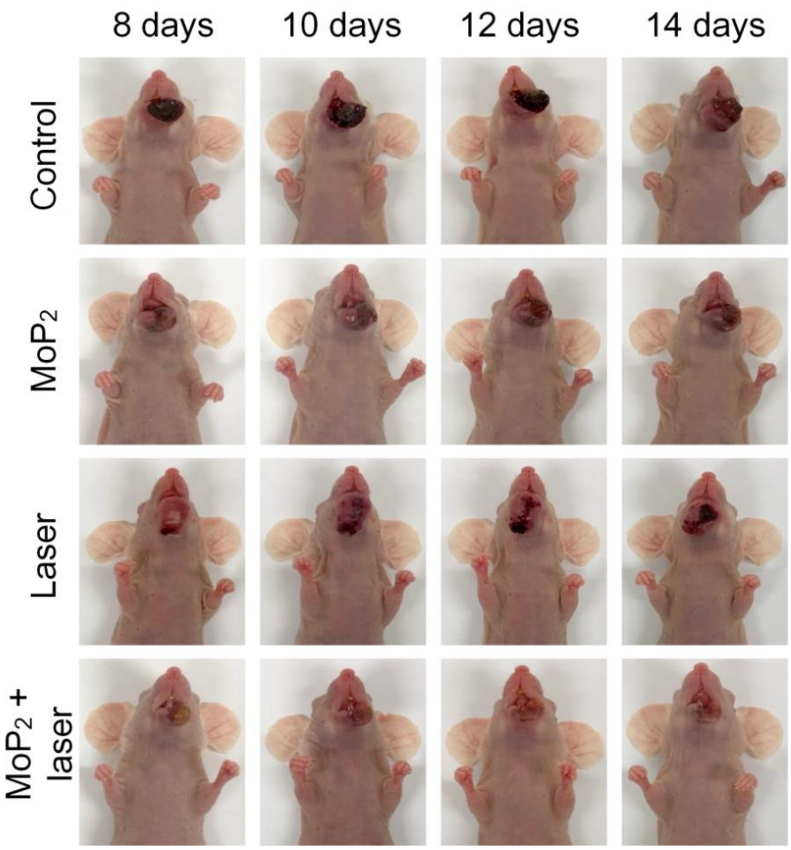


**Figure S18.** Photos of representative tumor volume variations treated with PBS, $MoP_2$ NRs, laser, and $MoP_2$ + laser at 8-14 days.

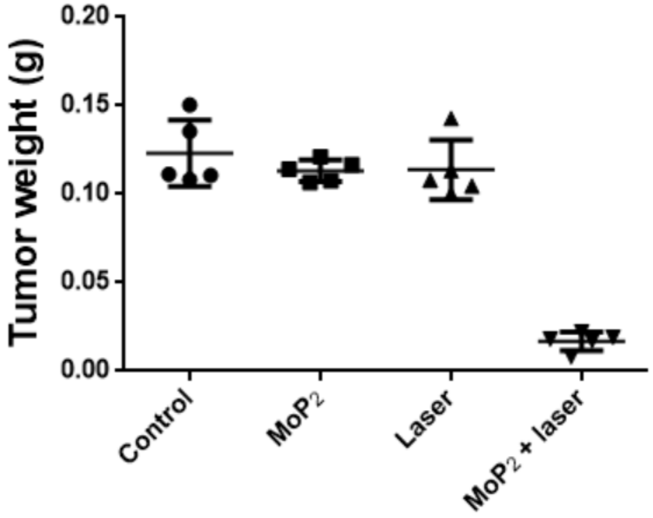


**Figure S19.** The average tumor weight of mice in each group at 14 days after receiving various treatments.

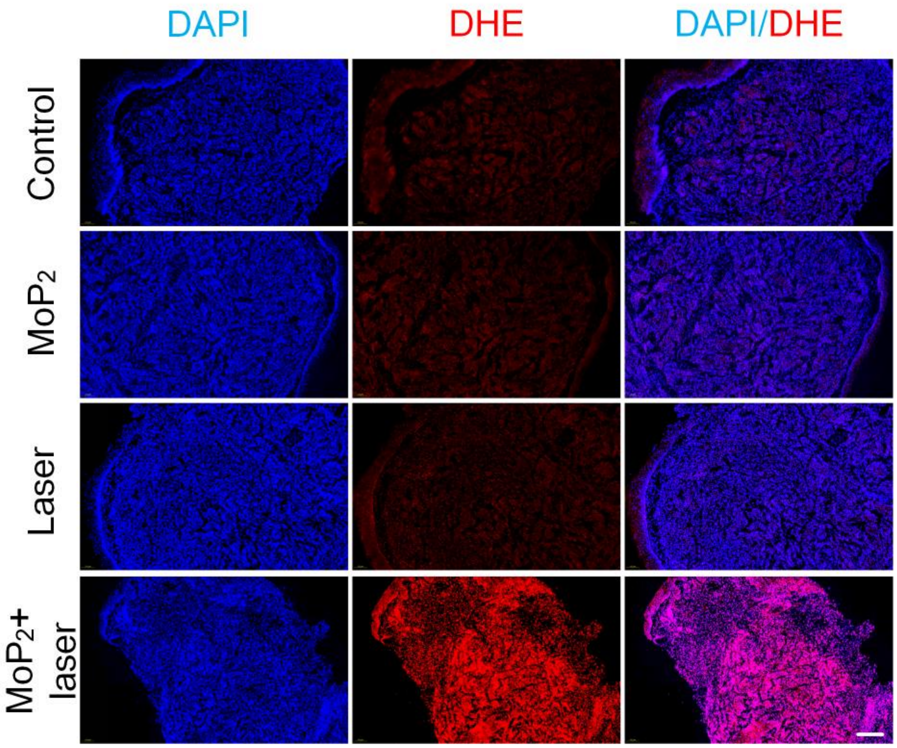


**Figure S20.** Tumor ROS analysis *via* DHE assay by fluorescence microscopy in CAL27 tumor tissues after different treatments (scale bar: 200 μm).

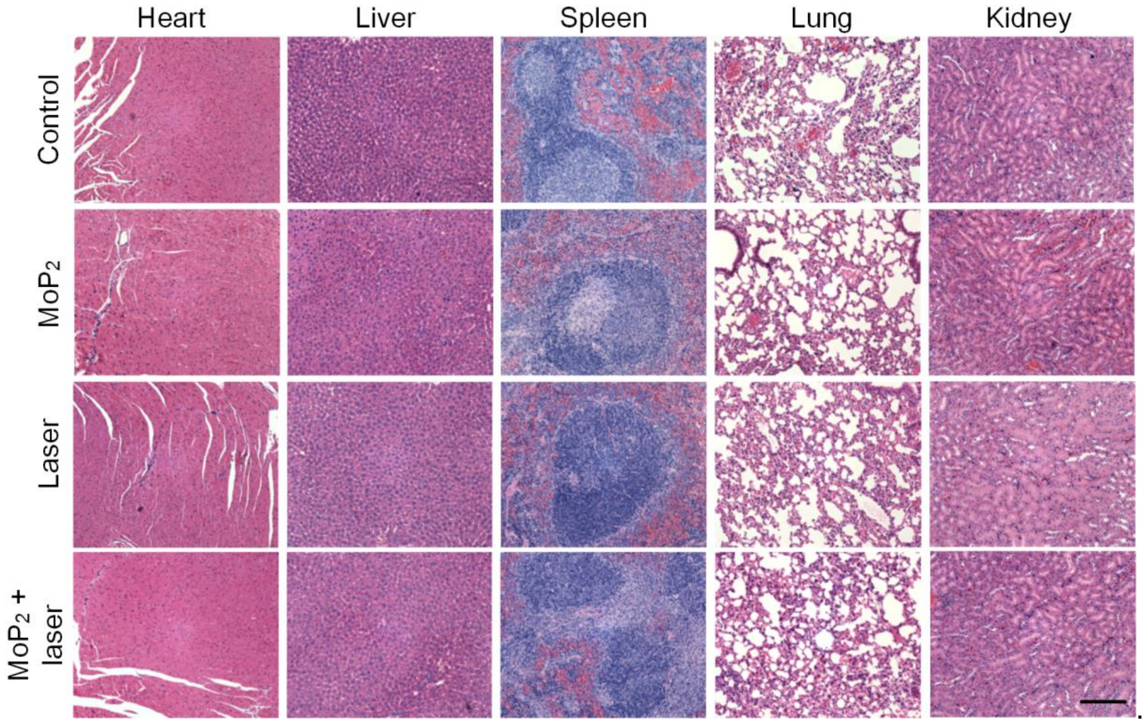


**Figure S21.** *In vivo* toxicity assessment. H&E stained images obtained from the liver, spleen, kidney, heart and lung of the mice at 14 days after receiving various treatments (scale bars, 100 µm for all panels).

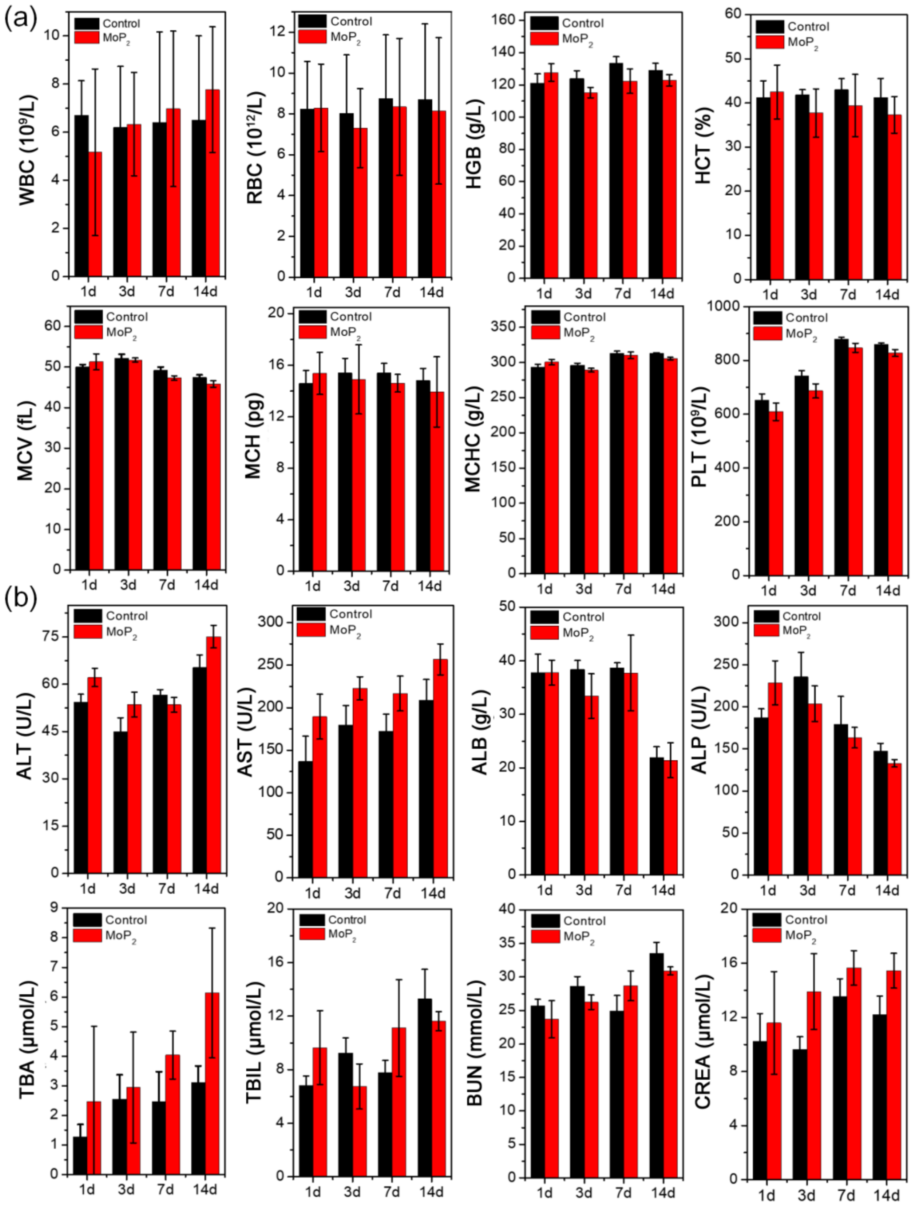


**Figure S22.** *In vivo* toxicity evaluation. (a) blood hematology and (b) biochemistry parameters of the mice treated with $MoP_2$ NRs(8 mg $kg^{-1}$) at the different time points of 1, 3, 7, and 14 days.

## References:


[1] W. Liu, X. S. Li, W. T. Li, Q. Q. Zhang, H. Bai, J. F. Li, G. C. Xi, *Biomaterials*. **2018**, *163*, 43.

[2] Q. Q. Yu, Y.M. Han, X. C. Wang, C. Qin, D. Zhai, Z.F. Yi, J. Chang, Y. Xiao, C. T. Wu, *ACS Nano*. **2018**, *12*, 2695.

[3] X. X. Han, Y. Xu, Y. Y. Li, X. Zhao, Y. L. Zhang, H. Min, Y. Q. Qi, G. J. Anderson, L. H. You, Y. L. Zhao, G. J. Nie, *ACS Nano.* **2019**, *13*, 4379.
